# Supplementary material for: Eliciting women’s preferences for place of child birth at a peri-urban setting in Nairobi, Kenya: A discrete choice experiment
Source: PLoS One. 2020 Dec 10;15(12):e0242149. doi: 10.1371/journal.pone.0242149 (PMC7728449; doi:10.1371/journal.pone.0242149)
Supplement: S1 Appendix — (DOCX) [file pone.0242149.s001.docx]

**S1 Appendix 1. The Characteristics of women interviewed in the Focus Group Discussions**

| **Variable** | **Peri-urban setting** |
| --- | --- |
| Age (Years): mean (SD) | 22(6.6) |
| Age of children-months (SD) | 1.7 (0.5) |
| **Parity N (%)** |  |
| Primiparous | 14 (35) |
| Multiparous | 26 (65) |
| **Delivery facility N (%)** |  |
| Public level 4 sub-County health facility | 9 (22.5) |
| Public level 2 and 3 primary health  facility | 10 (25) |
| Mission health facility | 9 (23) |
| Private facility | 12 (30) |
| **Total (%)** | **40 (43)** |
