# Supplementary material for: Eliciting women’s preferences for place of child birth at a peri-urban setting in Nairobi, Kenya: A discrete choice experiment
Source: PLoS One. 2020 Dec 10;15(12):e0242149. doi: 10.1371/journal.pone.0242149 (PMC7728449; doi:10.1371/journal.pone.0242149)
Supplement: S3 Appendix — (DOCX) [file pone.0242149.s003.docx]

## **Example of a scenario in a choice-set card that was presented to the women**

| THE DISCRETE CHOICE EXPERIMENT ON ATTRIBUTES FOR PLACE OF DELIVERY in EMBAKASI-NORTH SUB COUNTY KENYA | | | |
| --- | --- | --- | --- |
| Our objective is to conduct a DCE experiment to explore the relative importance of attributes of place of delivery to Kenyan women living in the peri-urban setting of Embakasi-North in Nairobi County to try and elucidate what women’s value and their preferences are when they are making choices on place of delivery. You will be provided with a script on a mobile phone and you will be asked to imagine that you are pregnant and you are given a choice between the following two health facilities to deliver your baby in. Which one would you prefer? Facility A or Facility B? You also have an option of choosing none of the two health facilities as Option C. This implies delivering your baby at home. There are no right or wrong answers | | | |
| SAMPLE CHOICE CARD | | | |
| Attribute | Health Facility A | Health Facility B | Option C |
| Quality of clinical care during delivery | Good quality | Bad quality | (None of the two health facilities- home delivery) |
| Attitude of healthcare workers | Kind and supportive attitude | Unkind attitude |  |
| Cost of delivery services | 3000Ksh | 5000Ksh |  |
| Availability of equipment and supplies | Equipment supplies not available | Equipment & supplies available |  |
| Distance to health facility | Facility is close to home | Facility is far from home |  |
| Cleanliness of the health facility | A clean health facility | A dirty health facility |  |
| Your choice (tick only one) | □ | □ | □ |
