# Supplementary material for: Eliciting women’s preferences for place of child birth at a peri-urban setting in Nairobi, Kenya: A discrete choice experiment
Source: PLoS One. 2020 Dec 10;15(12):e0242149. doi: 10.1371/journal.pone.0242149 (PMC7728449; doi:10.1371/journal.pone.0242149)
Supplement: S4 Appendix — (PDF) [file pone.0242149.s004.pdf]

A DISCRETE CHOICE EXPERIMENT(DCE) ON PREFERENCE FOR PLACE OF DELIVERY IN EMBAKASI-NORTH SUB COUNTY

Improving maternal health has been a key commitment of the Kenyan Government through the Ministry of Health and County Governments. In 2013 a Free Maternity Services policy was initiated by a presidential decree to provide free delivery care and subsequently reduce barriers associated with receiving maternal health services.Strathmore University Institute for healthcare Management is conducting research to evaluate utilization of healthcare facilities to attempt to determine women's preferences for place of delivery using a Discrete choice experiment methodology

This experiment is been conducted from women's perspective and your household has been randomly selected to participate in this survey. We want to know your preferences for place of delivery. There are no right or wrong answers. While your response is extremely important to us, your participation is voluntary. This questionnaire will take between 10 to 15 minutes to complete. Your responses will be confidential and will not be linked to you or shared with anyone.

For any question about the research kindly contact:

Jackline Aridi, Phone 0715961081; Email: Jackline.Aridi@strathmore.edu

OR Prof. Gilbert Kokwaro, Director, Institute for Healthcare Management, Strathmore Business School, Strathmore University, Phone: 0722323651 Email :gkokwaro@strathmore.edu or Dr. Francis Wafula, Tel. No: 0722679467: E-mail: fwafula@Strathmore.edu. Dr Mary. B Adams. Tel No0739579629 marybadams@gmail.com

II.THE DISCRETE CHOICE EXPERIMENT ON PLACE OF DELIVERY

Our objective is to conduct a DCE experiment to explore the relative importance of health service characteristics to Kenyan women living in Naivasha sub-County, Nakuru County. This will be compared to a similar cohort of women in Embakasi North in Nairobi County to try and elucidate what women's preferences are when they are selecting a place of delivery.

You will be provided with a script (on a mobile phone/tablet) and you will be asked to imagine that you are pregnant and you are given a choice between the following two health facilities to deliver your baby in. Which one would you prefer? Facility A or Facility B? you also have an option of choosing none of the two health facilities as Option C. This implies delivering your baby at home. There are no right or wrong answers

The definition of attributes

1. Quality of clinical care at the health facility- is defined as how one is handled or received at the health facility by auxiliary health staff and health care providers. It includes elements of communication and service delivery.
    a. Good quality of clinical care at the health facility is treatment that lives women with a good facility-based experience
    b. Bad quality of clinical care at the health facility is treatment that lives women with a bad facility-based experience.

2. Attitude of health care workers- is defined as how you felt when you interacted with the health care worker and whether you felt that they supported you during labor and delivery
    a. Kind and supportive health care workers live women with a positive childbirth experience.
    b. Unkind and unsupportive health care workers live women with a negative childbirth experience.
3. Availability of medical equipment and drug supplies – is defined as whether you believed that medical equipment with a well-functioning theatre and incubators for pre-mature babies and other key equipment such as resuscitators were available. Drug supplies availability was defined as been able to be provided with key medication within the health facility grounds and not required to purchase them externally.
    a. Available medical equipment was when the aforementioned equipment had been seen at the health facility or other women's experience informed women. Drug supplies availability was also confirmed from word of mouth experiences of whether drugs were provided within the health facility
    b. Not available was defined as when a health facility was perceived as having a theatre that is not well equipped with the aforementioned equipment. Drug supplies are also purchased externally from the health facility
4. Distance to the health facility. Since most health facilities are within a 5km radius to women's residences within a peri-urban setting. Distance was defined as the time taken to reach the health facility using transportation (mostly public transportation as the women in this area report not owning personal vehicles)
    a. Short distance- was defined as a health facility within one hour in public transportation
    b. Long distance – was defined as a health facility more than one hour in public transportation

5. Availability of referral services. This was defined as the presence of an ambulance that could transfer a woman to a higher level of care in the event of an obstetric emergency. It also referred to the availability of accompanying health personnel in the ambulance to ensure that the woman was attended to during the emergency enroute to the higher level of care.
    a. Availability of referral services – was defined as the ability of the health facility to have an ambulance and supporting personnel
    b. Unavailability of referral services – health facilities that did not have their own ambulances and lack of supporting personnel to accompany women
6. Cost of delivery services. This was defined using the three most common charges for delivery services- ranging from the lowest to the highest respectively (3000Ksh, 5000Ksh and 8000Ksh).

SAMPLE CHOICE CARD

**Attribute**	Health Facility A	Health Facility B	Option C
**Interpersonal treatment at the health facility**	Good interpersonal treatment at the health facility	Bad interpersonal statement at the health facility	(None of the two health facilities/ Birth at home)
**Attitude of healthcare workers**	Kind and supportive attitude	Unkind and unsupportive attitude	
**Cost of delivery services**	3000Ksh	5000Ksh	
**Availability of equipment and supplies**	Medical equipment& drug supplies not available	Medical equipment & drug supplies available	
**Distance to health facility**	Facility is close to home (less than hour travel time)	Facility is far from home<br>(more than one hour travel time)	
**Availability of referral services at health facility**	Referral services not available (ambulance services not available to the next level health facility)	Referral services available such as ambulance to the next level health facility	
**Your choice (tick only one)**	□	□	□
**How certain are you about this choice?**<br>**Not Certain □ Slightly certain □ Moderately certain □ Certain □ Very certain □**			

You will see that each choice has different combination of attributes. In this example you can see Option A has good quality services, kind attitude , costing 3000Ksh and is close to home, it however lacks supplies & equipment and referral services. Option B has poor quality services, with unkind workers, is far from home and costs 5000ksh. However, it has equipment and supplies and referral services. Option C means no health facility / home delivery. You are required to make trade-offs with the attributes presented.

THANK YOU FOR YOUR HELP!
