# Supplementary material for: Eliciting women’s preferences for place of child birth at a peri-urban setting in Nairobi, Kenya: A discrete choice experiment
Source: PLoS One. 2020 Dec 10;15(12):e0242149. doi: 10.1371/journal.pone.0242149 (PMC7728449; doi:10.1371/journal.pone.0242149)
Supplement: S5 Appendix — (PDF) [file pone.0242149.s005.pdf]

Respondent	Choice task	Alternatives	Altertanative Specific Constant	I.	II.	III.	IV.	V.	VI.
			A	B	C	D	E	F	G
1	1	1	1	0	0	0	1	1	3000
1	1	2	1	1	1	1	1	0	5000
1	1	3	0	0	0	0	0	0	0
1	2	1	1	1	1	0	1	1	3000
1	2	2	1	1	0	0	0	0	3000
1	2	3	0	0	0	0	0	0	0
1	3	1	1	1	0	0	0	1	3000
1	3	2	1	0	1	1	1	0	5000
1	3	3	0	0	0	0	0	0	0
1	4	1	1	0	1	1	0	0	5000
1	4	2	1	1	0	0	1	1	8000
1	4	3	0	0	0	0	0	0	0
1	5	1	1	1	1	1	1	1	8000
1	5	2	1	0	0	0	0	0	5000
1	5	3	0	0	0	0	0	0	0
1	6	1	1	0	0	1	0	0	3000
1	6	2	1	1	1	1	1	1	5000
1	6	3	0	0	0	0	0	0	0
1	7	1	1	1	0	1	1	0	8000
1	7	2	1	1	1	0	0	1	3000
1	7	3	0	0	0	0	0	0	0
1	8	1	1	1	1	1	1	1	8000
1	8	2	1	0	0	0	1	1	5000
1	8	3	0	0	0	0	0	0	0
1	9	1	1	0	1	1	1	0	8000
1	9	2	1	1	0	0	0	1	3000
1	9	3	0	0	0	0	0	0	0
1	10	1	1	1	1	0	0	0	5000
1	10	2	1	0	0	1	1	1	8000
1	10	3	0	0	0	0	0	0	0
1	11	1	1	0	1	0	1	0	3000
1	11	2	1	1	0	1	0	1	5000
1	11	3	0	0	0	0	0	0	0
1	12	1	1	0	0	1	1	1	3000
1	12	2	1	1	1	0	0	0	5000
1	12	3	0	0	0	0	0	0	0
1	13	1	1	0	1	0	1	1	8000
1	13	2	1	1	0	1	0	0	5000
1	13	3	0	0	0	0	0	0	0
1	14	1	1	0	1	0	1	0	3000
1	14	2	1	1	0	1	0	1	3000

1	14	3	0	0	0	0	0	0	0
1	15	1	1	1	1	1	1	1	3000
1	15	2	1	0	0	0	0	1	3000
1	15	3	0	0	0	0	0	0	0
1	16	1	1	1	1	0	1	1	3000
1	16	2	1	0	0	1	0	1	3000
1	16	3	0	0	0	0	0	0	0
