# Supplementary material for: Eliciting women’s preferences for place of child birth at a peri-urban setting in Nairobi, Kenya: A discrete choice experiment
Source: PLoS One. 2020 Dec 10;15(12):e0242149. doi: 10.1371/journal.pone.0242149 (PMC7728449; doi:10.1371/journal.pone.0242149)
Supplement: S6 Appendix — (PDF) [file pone.0242149.s006.pdf]

Field	WOMEN'S HEALTH AND HOUSEHOLD QUESTIONAIRRE
**Consent**	1. Has the participant signed the consent form?
**Respondent type**	2. Is this a main respondent or additional respondent?<br>A. Main<br>B. Additional
**mainPhone**	3. What is the phone number of the main respondent?
**mainFirst**	4. What is the first name of the main respondent?
**mainLast**	5. What is the last name of the main respondent?
	Thank you for participating in our survey. I am now going to ask you some basic questions about yourself.
	**DEMOGRAPHIC QUESTIONS**
**firstName**	6. What is your first name?
**lastName**	7. What is your last name?
**Age**	8. What is your age?
**PhoneNumber**	9. What is your phone Number?
**Residence**	10. How long have you lived in Embakasi North/Naivasha sub county?<br>A. I have lived here my whole life<br>B. I just moved to Embakasi North/Naivasha<br>C. Other
**MovedWhen**	11. How many years ago did you move here?<br>A. 0-5 years<br>B. 5-10 years<br>C. 11-20 years<br>D. Over 20 years
**ResidenceWhy**	12. **Why did you move to Embakasi North?**<br>A. I have family or friends here<br>B. I heard there were business opportunities here<br>C. To be close to Nairobi City Centre<br>D. To look for work<br>**E. Other**
**Schooling**	13. What is your level of education?<br>A. Did not attend primary school<br>B. Primary School<br>C. Secondary School<br>D. Tertiary<br>E. University
**Married**	14. Are you married<br>A. No<br>**B.** yes
**MarriedDuration**	15. How long have you been married?<br>A. 0-5 years<br>B. 5-10 years<br>C. 10-15 years<br>D. 15-20 years

HOUSEHOLD MODULE

**HeadofHousehold(HoH)**	16. Are you the head of the household?(If an important decision is to be made in the Household are you the one who gets to decide<br>A. No<br>B. Yes
**HeadofHouseholdNO**	17. How are you related to the person who makes important decisions for your household?<br>A. My Father/ My Husband/Boyfriend's father<br>B. My grandfather/ My Husband/Boyfriend's grandfather<br>C. My husband/Boyfriend<br>D. My mother/My Husband/boyfriend's mother<br>E. Another family member/ relative/ aunt/uncle<br>F. Other
**HeadofHouseholdNOInfluence**	18. Do you feel like your opinion is taken into account when decisions are made for the household? Would the head of the household be willing to change their decision if you disagreed?<br>A. No<br>B. Yes
**Main Earner**	19. Are you the main earner in your household? Do you contribute the most to household expenditures?<br>A. No<br>B. Yes
**MainEarnerNo**	20. How are you related to the person who earns most in your household?<br>A. My Father/ My Husband/Boyfriend's father<br>B. My grandfather/ My Husband/Boyfriend's grandfather<br>C. My husband/Boyfriend<br>D. My mother/My Husband/boyfriend's mother<br>E. Another family member/ relative/ aunt/uncle<br>F. Other
**ToiletNo**	21. Where do you usually use the toilet?<br>A. Public Toilet<br>B. A friend's/Family dwelling<br>C. Nearby business<br>D. Outdoors<br>E. Other
**Food Prepare**	22. Do you regularly prepare meals for the people in your household?<br>A. No<br>B. Yes

HOUSEHOLD MODULE : SOCIOECONOMIC STATUS	
**HoHMESame**	23. Are the head of the household and the main earner the same person in your household?<br>A. No<br>B. Yes
**adults**	24. How many people over the age of 18 live in your household?
**adultswomen**	25. How many of these are women?
**adolescents**	26. How many people aged between 14 and 18 live in the household
**adolescentwomen**	27. How many of these people are women?
**children**	28. How many people under 13 live in your household?
**childWomen**	29. How many of these children are women?
**employedadults**	30. How many members of your household contributed to your household expenses last month? *(this includes things like rent, food, water, electricity fuel, cooking fuel)*
**totalpublicexpenditure**	31. How much did the employed adults contribute to your household expenses last month
**drinkingSource**	32. Where do you get your drinking water from(The water that you use for drinking but not for preparing tea)<br>A. We have a tap and running water<br>B. It is delivered in large quantities then we store it<br>C. We buy bottles/sachets of water<br>D. A public tap<br>E. From a friend/family member<br>F. The river<br>G. Other
**drinkingSourceDelivery**	33. How often do you get water delivered to you?<br>A. Once a day<br>B. Once every few days<br>C. Once a week<br>D. Once a month<br>E. Once every few months
**drinkingSourceDeliveryQuantity**	34. How many liters of water do you get per delivery
**drinkingSourceDeliveryPrice**	35. How much does a delivery cost?
**drinkingSourceStorage**	36. The water that gets delivered does it arrive in a container or do you store it yourself?<br>A. It arrives in its own container<br>B. I store it myself
**drinkingSourceSealed**	37. Is the container that you store water in sealed when you are not getting water?<br>A. No<br>B. Yes
**drinkingSourceDuration**	38. The water that gets delivered and stored, how many days do you usually keep it before getting rid of it?
**drinkingSourceClean**	39. How often do you clean the container where you store the water with soap and boiling water?

	A. Every delivery<br>B. Once every few deliveries<br>C. Once a month<br>D. Once every few months<br>E. rarely
**foodprepsource**	40. Do you use that water for preparing food?<br>A. No<br>B. Yes
**foodprepboil**	41. Water that doesn't come from a tap in a household or a sealed bottle or a sachet, do you boil it before drinking it or using it for cooking?<br>A. No<br>B. Yes
**foodprepHandwash**	42. The person who prepares the most meals in your house, does he/she wash their hands before preparing the food?<br>A. No<br>B. Yes
**foodprepHandwashSoap**	43. Does He/She use soap?<br>A. No<br>B. Yes
**foodprepHandwashWater**	44. Does the water for handwashing come from a tap, sealed bottle or sachet or is it boiled?<br>A. No<br>B. Yes
**toilet**	45. Do you have a toilet in your dwelling or building<br>A. No<br>B. Yes
**toiletConnected**	46. Is your toilet connected directly to a sewer network or does it empty into roadside drainage?<br>A. Sewer network<br>B. Roadside drainage<br>C. Other
	**HOUSEHOLD ASSETS**
**Refrigerators**	47. How many refrigerators does your household own?
**Bicycles**	48. How many bicycles does your household own?
**Motorbikes**	49. How many motorbikes does your household own?
**Cars**	50. How many cars does your household own?
**Televisions**	51. How many Televisions does your household own?
**Radios**	52. How many radios does your household own?
**Stereos**	53. How many stereos does your household own?
**Mobiles**	54. How many mobiles does your household own?
**Laptops**	55. How many laptops does your household own?
**Fans**	56. How many fans does your household own?
**Generators**	57. How many generators does your household own?
**Mattresses**	58. How many mattresses does your household own?
**Air conditioners**	59. How many air conditioners does your household own?

**waterExp**	60. How much did your household spend on water last month?
**electricityExp**	61. How much did your household spend on electricity last month?
**fuelExp**	62. How much did your household spend on fuel last month?
**schoolfees**	63. In the last year, has your household been unable to afford fees for one of the schools at which a child is enrolled?<br>A. No<br>B. Yes
**foodSecurity**	64. In the last year, has there been a time where your household could not afford enough food for everyone to eat until they were no longer hungry?<br>A. No<br>B. Yes
**cooking**	65. When you cook what fuel did you use to warm up the food?<br>A. Paraffin<br>B. Charcoal<br>C. Gas<br>D. Electric Heater<br>E. Other
**cookingtimes**	66. How many times a day do you cook in a day?<br>A. 0<br>B. 1-2<br>C. 2-3<br>D. 3-4
**smokers**	67. Does anyone smoke inside your household?<br>A. No<br>B. Yes
**smokingtimes**	68. How many times a day someone smokes inside the house?

MOTHERHOOD AND FERTILITY MODULE

**insurance**	69. Do you currently have health insurance?<br>A. No<br>B. Yes
**insuranceType**	70. What kind of insurance do you have?<br>A. NHIF<br>B. OBA<br>C. Private Insurance<br>D. Other
**insuranceOther**	71. The private insurance policy you have, what is the name of the company that provides it? specify
**insurancePrice**	72. How much do you pay per month for insurance?<br>(if the respondent doesn't pay monthly help them approximate the monthly rate)
**generalHospital**	73. Have you visited a clinic, hospital, or doctor in the last year to receive medical care unrelated to a pregnancy?<br>A. No<br>B. Yes

**generalHospitalWhy**	74. During the most expensive visit to a clinic, hospital, or doctor in the last year, what was the visit for?<br>A. I was hurt in an accident and needed urgent care (example broken bones, stitches, allergic actions)<br>B. I was very sick and needed to get medicine or another kind of treatment example malaria, pneumonia)<br>C. I developed a condition and needed to sneak
**generalHospitalPaid**	75. How much did you spend in total on medical care received in the last year unrelated to pregnancy**?**
**generalHospitalStill**	76. Are you still seeking treatment for health conditions unrelated to pregnancy?<br>A. No<br>B. Yes
**anaemia**	77. Do you suffer from anemia?
**pregnantEver**	78. Have you ever been pregnant?
**pregnantAvoid**	79. Have you ever used anything or tried in any way to delay or avoid getting pregnant?
**rankFacilities**	80. **Facility Ranking**<br>I am going to list some facilities where you could give birth. Imagine that all costs of the delivery were covered, included bed fees and other expenses. Please rank them in order of your preferences for where you would deliver, where 1 is your preferred choice, 2 is your second choice, and so on, up to your ninth choice. If you don't recognize the name of a hospital, please tell me that information. If you would refuse to give birth there under any circumstances please tell me that as well.
**kenyattaRank**	81. Kenyatta National Hospital
**marysRank**	82. St Mary's Hospital
**mamalucyRank**	83. Mama Lucy's Hospital
**pumwaniRank**	84. Pumwani Maternity Hospital Rank
**uhaiNeemaRank**	85. Uhai Neema Hospital
**samaritanRank**	86. Samaritan Hospital
**mkungaRank**	87. Mkunga Rank
**paradiseRank**	88. Paradise
**provideRank**	89. Provide International
**timesPregnant**	90. How many times have you been pregnant?
**livebirths**	91. How many livebirths have you had?
**deaths**	92. Sometimes it happens that children die. It may be painful to talk about and I am sorry to ask you about such memories, but

	it is important to get correct information. Have you ever given birth to a son or daughter who was born alive but later died?
**deathsBoys**	93. How many of those were boys?
**miscarriages**	94. How many times have you had a pregnancy result in a miscarriage?
**stillbirths**	95. How many times have you had a pregnancy result in a stillbirth?
**yearPreg**	96. In what year did this pregnancy occur?
**embakasinorthPreg**	97. Were you living in Embakasi North sub County during this pregnancy?
**nairobiPreg**	98. Were you living in Nairobi during this pregnancy?
**intended**	99. Was this pregnancy planned?
**marriedThen**	100. Were you married to the father at the time?
**monthsPreg**	101. How many months were you pregnant before you gave birth?
**twins**	102. How many children were you carrying?
**workedThatYear**	103. Had you held a job or done any work outside the household in the year before giving birth?
**weeksBefore**	104. How many weeks before the birth did you stop working?
**antenatalcare**	105. How many ante natal visits did you attend?
**anc_first**	106. How many months pregnant were you when you first went for an ante natal care visit?
**ancSame**	107. Did you get ante natal care at the same facility where you planned to give birth?<br>A. No<br>B. Yes
**ancElseWhy**	108. Why did you go somewhere different for ante natal care than the place you planned to give birth?
**ancElseWhyMain**	109. What was the main reason you when somewhere different for ante natal care than the place you planned to give birth?<br>A. I was saving up to give birth in a nicer hospital than where I received ante natal care<br>B. I could afford ante natal care at that hospital, but not a birth there<br>C. Convenience: it was easier to go to the place where I received ante natal care than where I gave birth<br>D. Complications: I needed to go to a special hospital like Kenyatta because of complications

**insurancePr**	110. Did you have health insurance during this pregnancy?
**insuranceTypePr**	111. What kind of insurance did you have?
**insurancePricePr**	112. How much did you pay per month for insurance?
**talkPrice**	113. Did anyone talk to you about how expensive it would be to give birth during ante-natal care?
**contactHospital**	114. Did you contact hospitals about prices before giving birth?
**savingMonths**	115. How many months before you gave birth did you begin putting aside money to pay for it?
**iron**	116. During this pregnancy, did you take any iron tablets or iron syrup?
**Folic acid**	117. During this pregnancy, did you take any folic acid?
**malarial**	118. During this pregnancy, did you take any anti-malarial medication?
**tetanus**	119. During this pregnancy, did you receive a shot in the arm to prevent the baby from getting tetanus (convulsions after birth)?
**vitA**	120. Did you experience any problems seeing during the daytime or at night?
**smokeDuring**	121. Did you regularly smoke during this pregnancy?
**drinkDuring**	122. Did you regularly drink during this pregnancy?
**choresDuring**	123. During this pregnancy, who did the household chores like cooking and cleaning?
**specialist**	124. Did you visit an OB/GNY or specialist before giving birth?<br>A. No<br>B. Yes
**referral**	125. Were you referred to a larger hospital like Kenyatta National because a doctor determined that there might be complications with your pregnancy?<br>A. No<br>B. Yes
**complicationExpected**	126. What complication where you referred for?<br>A. Sepsis<br>B. Hemorrhage<br>C. High blood pressure<br>D. Other
**whereBirth**	127. Where did you give birth on this occasion?<br>A. Hospital<br>B. home
**plannedFacility**	128. Is this where you originally planned to give birth, or did you have to change your plans?<br>A. No<br>B. Yes
**whyChangePlans**	129. Why did you change your plans?<br>A. The baby came early and I had to go to the nearest facility<br>B. I wasn’t able to afford the facility I originally planned on

	C. I had more money that I expected when the baby was born so I could go to a nicer facility<br>D. Other
**outsideFacility**	130. Why didn't you deliver in a health facility?<br>A. It was too expensive<br>B. I couldn't get to one in time once I went into labor<br>C. I don't trust the Doctor and nurses at the facilities I can afford<br>D. I don't trust health facilities<br>E. Other
**whyHere**	131. What qualities of the Health Facility did you find important in making the choice of delivering there?<br>A. Cost<br>B. Cleanliness<br>C. Distance from home<br>D. Availability of supplies and equipment<br>E. Qualification of health worker(nurse or doctor)<br>F. Waiting time<br>G. Staff attitude<br>H. Referral by relative<br>I. Other
**whyHereMost**	132. What was the most important quality of the Health Facility in making the choice of delivering there?<br>A. Cost<br>B. Cleanliness<br>C. Distance from home<br>D. Availability of supplies and equipment<br>E. Qualification of health worker(nurse or doctor)<br>F. Waiting time<br>G. Staff attitude<br>H. Referral by relative<br>I. Other
**birthTime**	133. About how many hours did it take to deliver the baby, starting from when you first experienced contraction pains?
**ceaserean**	134. Was this a normal birth, or was the baby delivered by cesarean section?<br>A. Normal birth<br>B. Ceaserean
**ceasereanEmergency**	135. Was the cesarean planned or unexpected?<br>A. Planned<br>B. Unexpected
**doctorAtAll**	136. After you arrived at the hospital to give birth, did you see a doctor, or only nurses and birth attendants?<br>A. No<br>B. Yes
**doctorPresent**	137. Was a doctor present at the hospital when you gave birth?<br>A. No<br>B. Yes
**birthweight**	138. How many kilograms did the baby weigh at birth?

**babyGender**	139. Was the baby a boy or girl?<br>A. Male<br>B. Female
**BabiesGender**	140. How many of the babies were boys?
**pricePaid**	141. In total how much did you pay for the birth at the hospital including bed fees
**priceDebt**	142. Were you prepared to pay that much, or did you have to pay it back over time?<br>A. No<br>B. Yes
**priceExpect**	143. Did you expect a lower price for the birth before you received the bill?<br>A. No<br>B. Yes
**priceDebtpaid**	Have you paid off the bill from this pregnancy?<br>A. No<br>B. yes
**complicationRealised**	144. Did you experience any of the following complications when you gave birth on this occasion?<br>A. Sepsis<br>B. Hemorrhage<br>C. High blood pressure<br>D. Other
**Violence**	145. Did you experience any of the following while at the hospital?<br>A. Rude nurses<br>B. Requests for water were refused<br>C. Left alone for long periods of time or ignored<br>D. Yelled at by doctors or nurses<br>E. Pain or bleeding was ignored by Doctors or nurses
**daysHospital**	146. How many days did you stay in the hospital after giving birth?
**bleeding**	147. After returning home from the hospital, did you have any additional bleeding unrelated to menstruation in the following two months?<br>A. No<br>B. Yes
**return**	Did you have to return to the hospital to seek treatment?<br>A. No<br>B. Yes
**Satisfaction**	148. On a scale of 1 to 10, 1 being the best and 10 being the worst, how satisfied were you with the hospital at which you delivered on this occasion?
**recoveryTime**	149. How many weeks after the birth was it before you returned to normal activity?

**moreCare**	Did you receive additional care in the six months following the birth related to that pregnancy?<br>A. No<br>B. yes
**returnToWork**	150. How many weeks after the birth was it before you returned to work?
**twinsAlive**	151. Are all the children from this birth still alive?<br>A. No<br>B. Yes
**childAlive**	152. Is this child still alive?<br>A. No<br>B. Yes
**twinsStillAlive**	How many of the children are still alive?
**timeAlive**	153. How many months did the child live after giving birth?
**breastfeed**	154. Did you breastfeed the child?<br>A. No<br>B. Yes
**reside**	155. Does this child reside with you currently?
**resideWhere**	156. Where does this child currently live?
**diarrhea**	157. Has the child had diarrhea in the last two weeks?<br>A. No<br>B. Yes
**diarrheaBlood**	158. Was there any blood in the stool?<br>A. No<br>B. Yes
**pregnantNow**	159. Are you currently pregnant?<br>A. No<br>B. Yes
**totalDesiredFertility**	160. How many children do you want to have in total?
**pregnancydesire**	161. Would you like to become pregnant in the next two years?
**FamilyPlanning**	162. Are you currently using any family planning or contraception methods?
**contraceptionMethods**	163. What methods are you using<br>A. Rhythm method<br>B. Condoms<br>C. Pills<br>D. Intra-uterine device(IUD)<br>E. Withdrawal<br>F. Other
	Thank you for participating in our survey. We really appreciate your time, and are grateful for meeting with us today.
