# Supplementary figures and images for: Eliciting women’s preferences for place of child birth at a peri-urban setting in Nairobi, Kenya: A discrete choice experiment
Source: PLoS One. 2020 Dec 10;15(12):e0242149. doi: 10.1371/journal.pone.0242149 (PMC7728449; doi:10.1371/journal.pone.0242149)

**S7 APPENDIX: SAMPLING MAP OF EMBAKASI-NORTH SHOWING GEOGRAPHIC POSITIONING SYSTEMS**


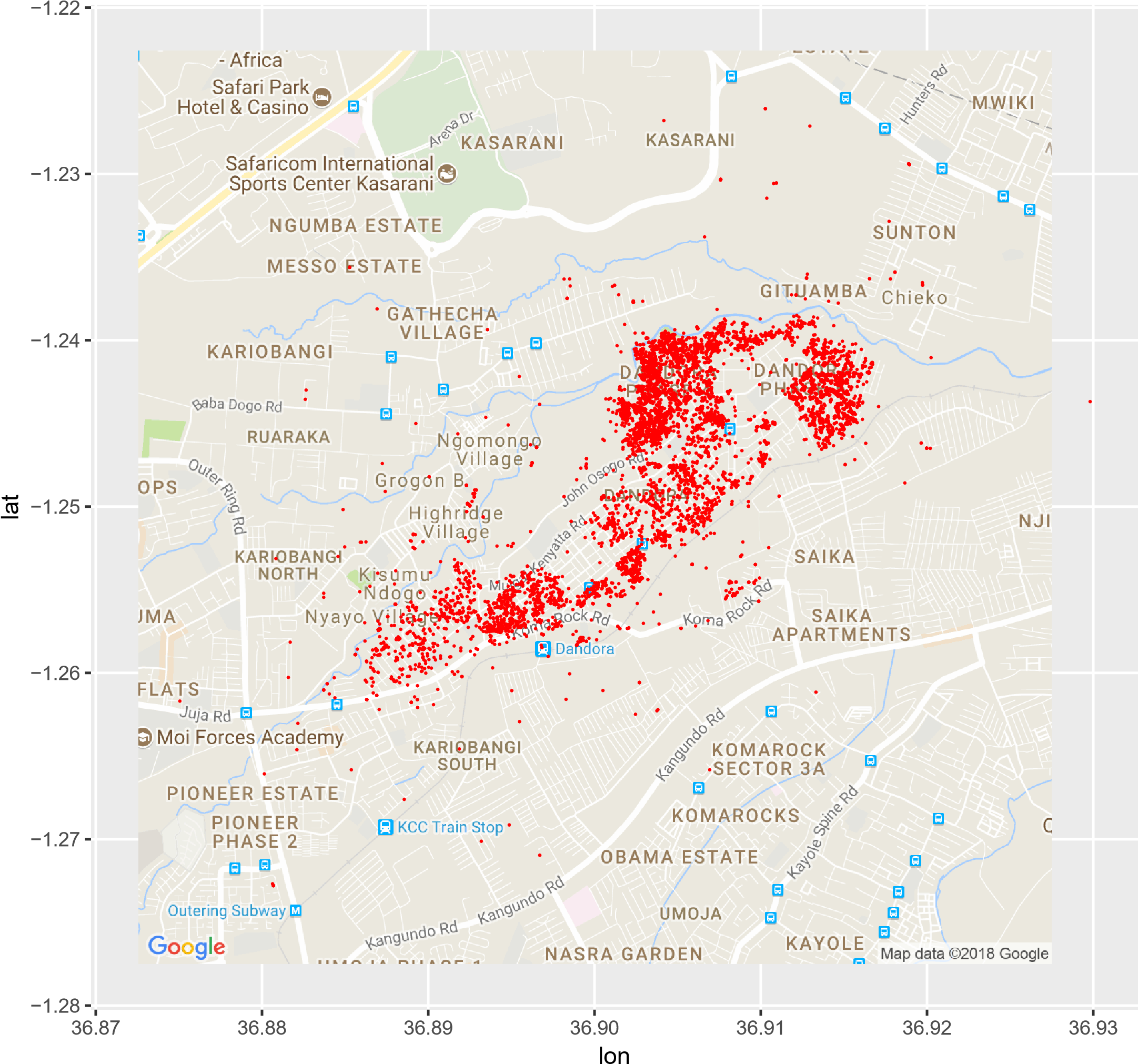

Supplement: S7 Appendix — (DOCX) [file pone.0242149.s007.docx]

**Digital presentation of the DCE experiment on Open Data Kit for women**


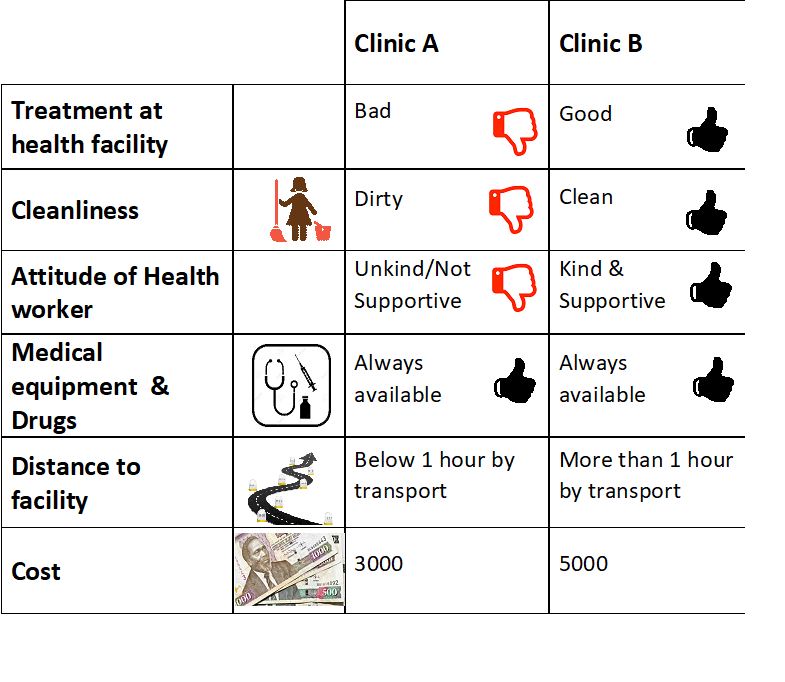

Supplement: S8 Appendix — (DOCX) [file pone.0242149.s008.docx]
