# Supplementary material for: Eliciting women’s preferences for place of child birth at a peri-urban setting in Nairobi, Kenya: A discrete choice experiment
Source: PLoS One. 2020 Dec 10;15(12):e0242149. doi: 10.1371/journal.pone.0242149 (PMC7728449; doi:10.1371/journal.pone.0242149)
Supplement: S11 Appendix — (PDF) [file pone.0242149.s011.pdf]

```
. clogit choice Qualityofclicservatdeliverygood cleanlinessclean attitudekindsupportive medicalequipmentdrugsavailable
>  DistanceShort cost asc_optout, group(ncs) robust

Iteration 0:   log pseudolikelihood = -3914.0522
Iteration 1:   log pseudolikelihood = -3416.0666
Iteration 2:   log pseudolikelihood = -3396.9778
Iteration 3:   log pseudolikelihood = -3396.8693
Iteration 4:   log pseudolikelihood = -3396.8693

Conditional (fixed-effects) logistic regression

                                                Number of obs     =     20,208
                                                Wald chi2(7)      =    4564.83
                                                Prob > chi2       =     0.0000
Log pseudolikelihood = -3396.8693               Pseudo R2         =     0.5410

                                                   (Std. Err. adjusted for clustering on ncs)
```

choice	Coef.	Robust Std. Err.	z	P>|z|	[95% Conf.	Interval]
Qualityofclicservatdeliverygood	.3856586	.0447454	8.62	0.000	.2979593	.4733579
cleanlinessclean	1.488392	.0434054	34.29	0.000	1.403319	1.573465
attitudekindsupportive	1.347663	.0383182	35.17	0.000	1.272561	1.422765
medicalequipmentdrugsavailable	1.435485	.0469386	30.58	0.000	1.343486	1.527483
DistanceShort	.3398182	.0368342	9.23	0.000	.2676246	.4120118
cost	.0002139	.0000135	15.83	0.000	.0001874	.0002404
asc_optout	1.424182	.1334776	10.67	0.000	1.162571	1.685794

```
. mixlogit choice, rand( Qualityofclicservatdeliverygood cleanlinessclean attitudekindsupportive medicalequipmentdrugs
> available DistanceShort asc_optout cost) ln(1) group(ncs) id(respondent_id) nrep(500) robust

Iteration 0:   log likelihood = -3371.0275  (not concave)
Iteration 1:   log likelihood = -3121.6697  (not concave)
Iteration 2:   log likelihood = -3067.1123  (not concave)
Iteration 3:   log likelihood = -2946.9284
Iteration 4:   log likelihood = -2839.4047
Iteration 5:   log likelihood = -2821.4347
Iteration 6:   log likelihood = -2815.9208
Iteration 7:   log likelihood = -2815.1069
Iteration 8:   log likelihood = -2813.6989
Iteration 9:   log likelihood = -2813.6941
Iteration 10:  log likelihood = -2813.6941

Mixed logit model                               Number of obs     =     20,208
                                                Wald chi2(7)      =   80646.83
Log likelihood = -2813.6941                     Prob > chi2       =     0.0000
```

choice	Coef.	Robust Std. Err.	z	P>|z|	[95% Conf.	Interval]
Mean						
Qualityofclicservatdeliverygood	.5709873	.0783975	7.28	0.000	.4173311	.7246435
cleanlinessclean	2.25872	.0916053	24.66	0.000	2.079177	2.438263
attitudekindsupportive	2.03907	.1249218	16.32	0.000	1.794227	2.283912
medicalequipmentdrugsavailable	2.26663	.0993085	22.82	0.000	2.071989	2.461271
DistanceShort	.4448705	.0291193	15.28	0.000	.3877977	.5019432
asc_optout	-2.008919	.5939098	-3.38	0.001	-3.172961	-.8448773
cost	-8.082067	.0544596	-148.40	0.000	-8.188806	-7.975329
SD						
Qualityofclicservatdeliverygood	.9264668	.0874638	10.59	0.000	.7550408	1.097893
cleanlinessclean	.5020036	.1128774	4.45	0.000	.2807679	.7232393
attitudekindsupportive	1.860955	.140606	13.24	0.000	1.585372	2.136537
medicalequipmentdrugsavailable	.5365863	.064391	8.33	0.000	.4103822	.6627904
DistanceShort	-.0051408	.0124837	-0.41	0.680	-.0296084	.0193269
asc_optout	5.023999	.49765	10.10	0.000	4.048623	5.999376
cost	-.0139806	.0530463	-0.26	0.792	-.1179495	.0899883

The sign of the estimated standard deviations is irrelevant: interpret them as being positive
