# Supplementary material for: Eliciting women’s preferences for place of child birth at a peri-urban setting in Nairobi, Kenya: A discrete choice experiment
Source: PLoS One. 2020 Dec 10;15(12):e0242149. doi: 10.1371/journal.pone.0242149 (PMC7728449; doi:10.1371/journal.pone.0242149)
Supplement: S12 Appendix — (DOCX) [file pone.0242149.s012.docx]

**The likelihood ratio (LR) test**

The likelihood ratio (LR) test is commonly used to evaluate the difference between nested models. One model is considered nested in another if the first model can be generated by imposing restrictions

On the parameters of the second. Most often the restriction is that the parameter is equal to zero. Hence does constraining the parameters to zero by leaving out the predictor variables significantly reduce the fit of the model. Hence we re-ran two models to assess the explanatory power for the models.

The log-likelihood ratio for the generalized mixed logit model without interactions was **-2813.69** it was co

mpared with the log-likelihood ration for the model with interactions. The model’s explanatory power was improved for most of the interactions (see highlighted) with the exception of the attributes related to attitude of health care worker.

| Interaction terms | Log Likelihood ratio for generalized mixed model with interactions |
| --- | --- |
| **Conditional logit model** | **-3396.87** |
| **Generalized mixed logit** | **-2786.00** |
| Clean_Age. | **-2845.93** |
| Clean_Marr | **-3067.24** |
| Clean_Main | **-3691.09** |
| Medequip_Age | **-3861.89** |
| Medequip_Marr | **-3835.91** |
| Medequip_Main | **-3834.97** |
| Att_Age | **-2801.22** |
| Att_Marr | **-2786.00** |
| Att_Main | **-2786.00** |
| QualClin_Age | **-3834.90** |
| QualClin_Marr | **-3837.18** |
| QualClin_Main | **-3836.93** |

The model’s explanatory power was improved for most of the interactions (see highlighted in Table 1 below) with the exception of the attributes related to attitude of health care worker. The LR test compares the log likelihoods of the two models and tests whether this difference is statistically significant. The LR test is significant as seen in the Stata Output below and the mixed model presents an improvement from the conditional logit model
