# Supplementary material for: Eliciting women’s preferences for place of child birth at a peri-urban setting in Nairobi, Kenya: A discrete choice experiment
Source: PLoS One. 2020 Dec 10;15(12):e0242149. doi: 10.1371/journal.pone.0242149 (PMC7728449; doi:10.1371/journal.pone.0242149)
Supplement: Supplementary file 10 [file pone.0242149.s009.docx]

```
. pwcorr Qualityofclicservatdeliverygood attitudekindsupportive, star(0.05) sig

             |  Quali~od attitu..
-------------+------------------
Qualityof~od |    1.0000
             |
             |
attitudeki~e |    0.2860*  1.0000
             |    0.0000
             |

.
. . pwcorr Qualityofclicservatdeliverybad attitudeunkindnotsupportive , star(0.05) sig

             |  Quali~ad a~unki~e
-------------+------------------
Qualityof~ad |    1.0000
             |
             |
attitudeun~e |    0.3080*  1.0000
             |    0.0000
             |
```
