# Supplementary material for: Eliciting women’s preferences for place of child birth at a peri-urban setting in Nairobi, Kenya: A discrete choice experiment
Source: PLoS One. 2020 Dec 10;15(12):e0242149. doi: 10.1371/journal.pone.0242149 (PMC7728449; doi:10.1371/journal.pone.0242149)
Supplement: Supplementary file 11 [file pone.0242149.s010.docx]

```
. tab facility_level_type
```

| facility_level_type | Freq. | Percent | Cum. |
|---|---|---|---|
| Private | 159 | 40.36 | 40.36 |
| Public | 235 | 59.64 | 100.00 |
| Total | 394 | 100.00 | |

```
. tab facility_level_withhome
```

| facility_level_withhome | Freq. | Percent | Cum. |
|---|---|---|---|
| Home | 27 | 6.41 | 6.41 |
| Tertiary | 318 | 75.53 | 81.95 |
| Primary | 76 | 18.05 | 100.00 |
| Total | 421 | 100.00 | |

```
. tab birthplace_facility_level
```

| birthplace_facility_level | Freq. | Percent | Cum. |
|---|---|---|---|
| Tertiary | 318 | 80.71 | 80.71 |
| Primary | 76 | 19.29 | 100.00 |
| Total | 394 | 100.00 | |
