# Supplementary material for: Eliciting women’s preferences for place of child birth at a peri-urban setting in Nairobi, Kenya: A discrete choice experiment
Source: PLoS One. 2020 Dec 10;15(12):e0242149. doi: 10.1371/journal.pone.0242149 (PMC7728449; doi:10.1371/journal.pone.0242149)
Supplement: Attachment — Submitted filename: RESPONSE TO REVIEWER_22ndFeb2020.docx [file pone.0242149.s014.docx]

**RESPONSE TO REVIEWER#1**

**Review Comments to the Author**

Please use the space provided to explain your answers to the questions above. You may also include additional comments for the author, including concerns about dual publication, research ethics, or publication ethics. (Please upload your review as an attachment if it exceeds 20,000 characters)

Reviewer #1: Thank you very much for the chance to review the paper by Ms. Aridi et al. on eliciting women’s preferences for a place of delivery in Kenya using a discrete choice experiment. Overall, the paper reports women’s preference and relative importance for a few key attributes and costs that women may consider when deciding a delivery place in a peri-urban setting in Kenya. The author presented interesting study design, analysis and results from a reasonably well-represented study population to address the authors’ research question. However, the paper has some methodological weaknesses, incomplete and unclear presentation of the data and analytical techniques, and questionable interpretation of the results, which significantly weaken the quality of the manuscript. There are a number of places where the sentences were poorly written, and lack of line numbers also made difficult to make comments.

**MAJOR COMMENTS**

**Study Design**

**Comment#1**

Although the authors indicated that they selected the attributes and attribute levels were selected based on a qualitative study, there are no description how a qualitative study was done. Please provide detailed information about the qualitative studies (the type of the qualitative study, how many interviews were done, brief sociodemographic characteristics of these women, and how these data were analyzed).

**Response to comment#1**

Thank you for your comment. A description of how the qualitative study was conducted has been added to the revised manuscript. The qualitative study was a phenomenological study and aimed to describe women’s experiences and perceptions of quality of care issues influencing their choice of a delivery facility. Six focus group discussions were done, one each, at 6 different facilities. These facilities represented the range of options available to women in this slum area. 40 women were purposively selected. The characteristics of the women interviewed as well as details on how the data was analyzed have been included in a draft manuscript attached as appendix 1.

**Comment #2**

I strongly think the two attributes, ‘Interpersonal treatment at the health facility’ and ‘Attitude of healthcare workers’ are correlated. How do these two attributes differ? Furthermore, I worry that the dichotomy of “good” vs” bad” interpersonal treatment seems too simplistic, and can be unclear and very subjective to respondents. Also, I suspect that’s one of the reasons why the authors see a much lower coefficient for the interpersonal treatment.

**Response to comment#2**

Your point is well taken and we appreciate the opportunity to explain.

In order to address your concerns around the attribute correlation we calculated the Pearson’s Products Moment (PPM) to assess for interattribute correlation. We found out that the Pearson’s correlation coefficient for the interattribute correlation between the attribute was between the attribute levels of QUALCLICGOOD (quality of clinical services good) and ATTITUDEKIND (attitude of healthcare worker kind) was 0.286. The correlation coefficient was 0.308 for the attribute levels of QUALCLICBAD (quality of clinical services good) and ATTITUDEUNKIND (unkindandunsupportive attitude of healthcare worker). Both were found to be insignificant at the 0.05 % level (See Appendix 2 with correlation matrix from Stata Output).

When women spoke about their experiences at the health facility the category we labelled ‘interpersonal treatment’ reflected two dimensions or aspects of care, (1) how the health facility handled them as clients/customers and (2) their perception of the clinical treatment they received during labor and delivery. This differs in important ways from the attribute labeled ‘attitude of the healthcare workers’ which was reflected descriptions of mistreatment/disrespect and abuse during delivery care. Disrespectful care is prevalent in low income settings where this study was conducted and there is extensive literature on the construct of disrespect/mistreatment of women during their delivery process. See (Bohren et al., 2014) ; (Oluoch-Aridi, Smith-Oka, Milan, & Dowd, 2018).

In our work we sought to capture the women’s voices. The women respondents identified a salient distinction between disrespectful care labeled ‘attitude of health care workers’ and the construct labeled ‘interpersonal treatment’.   Our priority was to hear and reflect the women’s voices in a way addressed the contextually relevant semantic meaning. These sentiments expressed in Swahili, the language we used to conduct the interviews, are distinct. In trying to express these two concepts in English- our choice of labels for the attributes insufficiently conveyed the distinction present in the Kiswahili. These two constructs are particularly important because are amenable to action by hospital management and policy makers. DCE’s are supposed to be focused on attributes that are policy amenable (Mangham, Hanson, & McPake, 2008).The attributes are present in the WHO framework and recognized in that they represent patient centered tenets of the experience of care (WHO, 2016). The attribute we labelled as interpersonal treatment in represented in the WHO framework as evidenced based

In saying this we acknowledge the important distinction between a lay persons understanding and articulation staff behavior as well as their limited understanding of what constitutes technical excellence.  To improve understanding of the attribute and its meaning, we have relabeled the attribute on interpersonal treatment at the health facility to ‘quality of clinical services during delivery’ with an aim to convey this distinction. We hope it relays a more accurate presentation of the women’s voices in English.

The distinction between our attribute labelled interpersonal treatment and the attribute labelled attitude of health workers in the manuscript has been labelled in other literature A similar study done in Zambia identifies attributes that are similar but distinct and important to policy makers namely (1) the likelihood that the hospital staff will examine the child properly, (2) staff attitudes (Hanson, McPake, Nakamba, & Archard, 2005).And the distinction is mathematically and we are sorry we didn’t label it well enough to identify the distinction in English

The identification of attributes levels within DCE’s is often simplistic. This is done to reduce the process of complex cognitive especially for women with lower education for ease of cognition and for the respondents to make choices (Mangham *et al*., 2008).This is consistent with other published literature

See,(Kruk, Paczkowski, Mbaruku, de Pinho, & Galea, 2009); (Kruk et al., 2010); (Larson et al., 2015))for similar types of attribute labels for DCE’s focused on place of delivery.

**Comment#3**

I think the biggest question for the DCE design is the attribute level of costs. Given that the authors say that the there are no delivery fees at public health facilities, why didn’t the authors include no cost as the level option? Are there any other associated fees women need to pay when they deliver at public health facilities? The authors did not provide a data on how many of these women had previously delivered at public health facilities, compared to private health facilities. If most of women deliver at public health facilities, I worry that the hypothetical scenarios of paying the extra money might have not been correctly judged by the respondents as designed. Also, I wonder how the authors chose the cut-offs for the costs (3000, 5000, 8000)? Please help the readers understand whether these are reasonable range of costs to deliver at the facilities (especially at the public health facilities).

**Response to comment#3**

In Kenya, there are several auxiliary costs associated with “free” delivery care. The rates of these auxiliary costs are variable and facility dependent, but they are pervasive. These costs may be clearly articulated at the facility - such as bed costs or charges for drugs or it may be just an arbitrary tax for delivery related materials such as diapers or cotton wool etc.

We did not include “no cost” as a level option because despite the free maternity whenever women go to delivery they are subjected to some cost to cover these expenses. That said, we do agree that the concept of auxiliary cost is not well articulated in the background section. The inception of “free maternity care” did substantially reduce out of pocket costs for women, but it did not eliminate them. We felt like giving the option of no cost would erroneously lead the women to make choices presuming they experienced no cost while in real life situations they are subjected to costs.

The cut-off for the costs were chosen using information provided by the women about how much they had spent as delivery costs in the public and private health facilities and both before and after the removal of user fees at public health facilities. We have included a paragraph in the methods section that shows that the cost estimates for delivery came from the qualitative study. For a review on how the implementation of the free maternity sometimes resulted in people paying fees see (Tama et al., 2018).

We do have data on the proportion of women who delivered at public health facilities compared to private health facilities and have included it in Table 5. 60% of the women delivered at public health facilities with 40% at private health facilities.( See Appendix 3 on Stata output on place of delivery)

**Comment#4**

The authors mentioned that they chose a random sample out of a larger household survey. Which household survey is this (please cite it and provide a brief description)? Also, please provide more description of how this random selection was done – was there any stratification by geographic region or any other characteristics? Was the survey used one fixed same questionnaire for everyone? Please specify.

**Response to comment#4**

The household survey tool was a composite that was based on the validated surveys; Kenya Demographic Health Survey (KDHS) 2014 (Kenya National Bureau of Statistics et al., 2015) and the African Population and Health Research Centers APHRC slum survey (2012) (APHRC), 2012). We used the questions that have been and added the APHRC slum survey to extend our understanding around economics

A paragraph in the methods section has been included to explain the sources of the survey questionnaire. Two citations have been provided to clarify where we obtained our survey from. We have also attached a copy of our household survey as an appendix in the main study. An explanation of the selection of participants into the study is explained below;

**Random selection for the main survey**

Dandora is only 4 square km and has a population of 151, 046 according to the 2009 census. The random selection of women in the survey was done by mapping out the Dandora area and generating 200 points on the geographic positioning system (GPS) from google earth. This file has been attached as Appendix 4 for the reviewer’s perusal.

After this a list of randomly generated numbers was created and matched to the 200 points in the map.  Starting from a selected point the enumerators were instructed to approach the households identified using the random numbers on the list.  The sample size calculation for the main survey was done using a formula based on the one used by the Slum Survey 2012. The following was the formula

n= Z2 1-α/2 p (1-p)

e2

n= the required sample size of the individuals in the target population

p= the expected rate or prevalence of the key indicator to be estimated. We used 61% of live births in the five years preceding the survey were delivered in a health facility (KDHS 2014)

Deff design effect - we used 1.5 (following APHRC (2012)

e= margin of error to be tolerated at 95 % level of confidence

Z = critical value for the standard normal distribution corresponding to a type 1 error rate of α

The household survey was administered to a total of 4640 randomly selected women. Then we used the criteria for the selection of the women for the DCE’s- this criteria was women who had delivered within one year at the time of the survey (October 2017). For the DCE a small proportion of roughly 10% of women who met the criteria of having delivered within one year and those who were aged between 18-49 years were selected to select the 481 women from the 4640 women.

**Comment#5**

Please give a detailed assumption and calculation for how the sample size was calculated using the de Dekker-Grob’s formula. What coefficients did the authors assume initially? Did the authors calculate based on the conditional logit model or mixed logit model for their initial sample size calculation? At what percent of power? What was the minimum sample size required for the study?

**Response to comment#5**

We appreciate your attention to detail and we have amended the manuscript to correctly state that we chose the Johnson and Orme method to calculate sample size, as opposed to Dekker-Grob’s formula. e (Johnson & Orme, 2003)

We used the rule by Johnson and Orme to suggest the sample size required for main effects depends on the number of choice tasks (t), the number of alternatives (a) and the number of analysis cells (c). The formula considers main effects C is equal to the largest number of levels for any of the attributes when considering all two way interactions. We anticipated two way interactions so we multiplied the largest level size 3 by 2=6

We had 16 Choice tasks (t) with 3 alternatives (a) and 3*2 analysis cells (c). We then used the formula by Johnson and Orme to calculate the sample size, as shown below:

= N> 500*c/t*a

= N> 500*6/16*3

=N> 62.5

We also looked at the Johnson and Orme article and it recommended that sample sizes 500 and above were a minimum threshold that were sufficient to estimate effects, this sample size remains higher than other sample sizes used in estimating DCEs Louivere (2010) Bliemer and Rose (2009) Our sample size targeted sample size of 481 and eventual response rate of 421 (87.5%) was large enough power to provide results that were statistically significant for all relevant attributes. We did not use the debekker Grob in our original estimation for the conditional logit versus the mixed logit however as you see from our data the sample size was sufficient to allow us to estimate and interpret the conditional logit and mixed logit model

**Comment #6**

Can the authors provide more details about the D-efficient design? Were orthogonality and level of balance achieved? How many times did each attribute level appear in the questionnaire? What was the goodness-of-fit for the test?

**Response to comment#6**

Design balance =A balanced design has each attribute level occurring equally often across all pairs of attributes (Mangham 2009). Our design showed that almost half of the attribute levels occurred 16 times (equally often)

Orthogonality = the number of times that each attribute levels appears. Orthogonality is a desirable property of experimental designs that requires strictly independent variation of levels across attributes, in which each attribute level appears an equal number of times in combination with all other attribute levels

We achieved a reasonable level of orthogonality and balance, the D-efficiency level. Below we document

- The alternative specific constant appears 16 times for the opt-out option and 16 times each for both the alternative A and B.
- For the attribute on the quality of clinical services during delivery- the attribute level good quality of clinical services during delivery appeared 19times while the attribute level bad quality of clinical services during delivery appeared 14 times.
- For the attribute on the cleanliness of the health facility- the attribute level of clean appeared 16 times and the attribute level of dirty appeared 16 times.
- For the attribute on kindness and supportive health worker the attribute on kind and supportive health worker appeared 16 times while unkind and unsupportive health worker appears 15 times
- For the attribute on availability of medical equipment and drugs, available appeared 18 times and unavailability of medical equipment and drugs appeared 14 times
- For the attribute on distance , short distance appeared 19 times and long distance appeared 13 times
- For the attribute on the cost of delivery services 3000Ksh appeared 15 times ad 5000Ksh appeared ten times and 8000 appeared 7 times

| **Attribute** | **Attribute Level** | **Coding** | **Number of times attribute level appears in the experimental design** |
| --- | --- | --- | --- |
| 1. Opt-out | Opt-out | 0 | 16 |
|  | Alternative A | 1 | 16 |
|  | Alternative B | 1 | 16 |
| 2. Quality of clinical delivery services | Good | 1 | 19 |
|  | Bad | 0 | 14 |
| 3. Cleanliness | Clean | 1 | 16 |
|  | Dirty | 0 | 16 |
| 4. Attitude of health care worker | Kind and supportive | 1 | 16 |
|  | Unkind and unsupportive | 0 | 15 |
| 5. Availability of medical equipment and drugs | Available | 1 | 18 |
|  | Unavailable | 0 | 14 |
| 6. Distance to the health facility | Short distance | 1 | 19 |
|  | Long distance | 0 | 13 |
| 7. Costs of delivery services | (In Kenya shillings) | 3000 | 15 |
|  |  | 5000 | 10 |
|  |  | 8000 | 7 |

**Comment #7**

- I am not sure why the authors would have blocked the questions into the two groups and ask each women answer two blocks anyway (it deficits the purpose of blocking…).

**Response to comment #7**

This is a semantic error and has been corrected to reflect the fact that women answered questions from one block each (8 questions each). This has been corrected to state, “…..The choice-sets were grouped into two, and each woman answered eight questions in a block…:”

**Comment#8**

- The authors indicated conducting a pilot study. Can the authors provide more details how the results from the pilot study testing were used to improve the study questionnaire?

**Response to comment#8**

The authors conducted a pilot study on a sample size of 30. In the pilot 6 attributes were selected for inclusion into the DCE. The pilot allowed us to text the most important and policy relevant attributes the coefficients that we found during the pilot were all significant even at the small sample size of 30, and hence we went ahead to conduct the experiment.

**Comment #9**

The formula for possible choices is incorrect: it should be “2^5 x 3^1 = 96” for two scenarios matched 96*95/2= 4560 possible choices in the full factorial

**Response to comment#9**

This formula has been corrected to reflect the correct number for the full fractional design.

**RESULTS SECTION**

**Comment#1**

The authors described that “opt-out” option was included in the model. How many people chose “opt-out” option out of all available choice tasks? Also, with about 26% of women planning not to deliver at health facilities, I worry whether there are other factors that influence women’s choices to deliver outside of health facilities (i.e. home). Did the authors look at preference coefficients among women who chose “opt-out” option at least once? Was there anyone who mostly chose the “opt-out” option?

**Response to comment#1**

Thank you for pointing this out, about one third of the women opted out of the DCE sequence.

On the 26% that you point above, the planned health facility was a variable constructed

but a response to the question on the variable on planned delivery health facility =Is this the health facility where you originally planned to give birth, or did you have to change your plans?

**Comment #2**

- Can the authors clarify the difference between Table 6 and 7?

**Response to Comment#2**

For increased clarity, the data was re-analyzed in Stata 15 with an aim to improve clarity. The limitation of the JMP software (SAS) does not allow stepwise regression to more clearly analyze and interpret the mixed logit model The original table 6 and 7 have been replaced with new tables 5 and 6 that address the results of the generalized mixed logit model and the mixed logit model with the interactions.

**Comment#3**

- I disagree with the approach that the authors put all possible combinations of different interactions between sociodemographic variables and attributes. Even so, I encourage not to show all the non-significant (especially if not driven by specific hypotheses) results in Table 6.

**Response to Comment#3**

We appreciate your comment and agree that the model might lead to hence significant bias. We re-analyzed the data with the interaction terms separately in Stata 15. We present two tables table 5 showing the mixed logit without interactions and a new table 6 that shows the model with interactions (that have been modelled individually)We have focused on sociodemographic variables that are likely to influence place of delivery in the literature such as age, marital status, main earner status.

**Comment #4**

What is log worth? The authors’ present acronym of the attributes which were not previously defined in the manuscript thus it is difficult to understand

**Response to Coment#4**

Log worth is language that is used by the software JMP to describe the magnitude of the coefficient of the parameter estimate. We have used STATA 15 software to re-analyze the data.

**Comment#5**

Table 6. Please present meaningful acronyms (then define them in the footnote) or full description of the attributes.

**Response to comment#5**

We have revised the acronyms of the attributes to be uniform throughout the manuscript and used easily understood nomenclature. A legend has been included at the end of the table 6

**Comment#6**

P-value should be presented up to 2-3 digits in the table and throughout the paper.

**Response to comment#6**

P-Values have been presented to 3 digits, this has been revised throughout the paper.

**Comment#7**

- I am surprised that the cost coefficient in the conditional logit model is positive. More confusingly, while the cost coefficient is positive in the conditional logit (Table 4), the cost coefficient in the mixed logit model is very significantly negative. Why is that?

**Response to comment#7**

The generalized mixed logit provides information on preference heterogeneity in the sample. The negative sign of the cost attribute for the mean parameter shows that on average women had a utility for lower cost. The positive sign in the conditional model suggests that the women had a utility for high costs. We agree that this might present some confusion on women’s actual preferences.

We attribute some of the weaknesses in the experimental design on the cost attribute might have demonstrated itself mathematically. Our experimental design that gave an unbalanced number of attribute levels for the attribute. We realized that the parameters might be biased and hence we will make our interpretations for the cost value cautiously. We have decided to focus on the other parameter estimates that were well specified.

Additionally these signs may also represent the confusion by the low income women in this setting on the actual costs of delivery and how to value them. The true cost is a difficult issue because the marginal cost were hidden in real life until after the delivery.

See quotation on confusion on costs from our qualitative study

*Costs of delivery services.* Overall costs were a major concern for women in both settings. This included direct costs related to financing the actual delivery service, but also indirect costs like transportation costs or opportunity costs associated with seeking delivery care. Sometimes additional costs were introduced inadvertently by some of the staff in public health facilities, leading to confusion and misunderstandings in health facilities that were advertised to be free. An example of a misunderstanding is illustrated below:

*"…To begin with, at the public hospital they told me that I had to pay 4,800 Ksh (48 USD) for the delivery service. My husband asked what it was for and they reduced it to 800 Ksh. He still insisted on knowing what the charges were for since he knew at a public health institution, maternity services were free of charge. In the end, he did not pay even a single cent…"*

*(FGD public tertiary health facility, rural setting)*

With 20,208 observations we had sufficient power to discern the cost value when there is rational correspond in the real world.

**Comment#8**

Table 6: It was not clear whether all interaction terms were fitted into one big model or whether each interaction was fitted separately). Please clarify. If all interaction terms were fitted, I would really worry about overfitting the model.

**Response to comment#8**

This has been addressed the authors have re-ran the mixed logit model again with interactions one at a time using Stepwise regression. We only focused on the sociodemographic attributes that have been proven to have an interaction with the attributes for place of delivery such as age, marital status main earner status. We have also only focused to show the significant and a few non-significant covariates for comparison. You can review this data in the new table 6.

**Comment#9**

Table 7: I had a hard time to understand and interpret this table. Can the authors present the log-likelihood ratio test where they compare the model without any interaction term, and the model with interaction terms with each of the sociodemographic variables?

**Response to comment#9**

The likelihood ratio (LR) test is commonly used to evaluate the difference between nested models. One model is considered nested in another if the first model can be generated by imposing restrictions

On the parameters of the second. Most often the restriction is that the parameter is equal to zero. Hence does constraining the parameters to zero by leaving out the predictor variables significantly reduce the fit of the model.

Hence we re-ran two models to assess the explanatory power for the models.

The log-likelihood ratio for the generalized mixed logit model without any interactions was **-2813.69** it was compared with the different log-likelihood ratios for the model with interactions. See Table 1 below.

The model’s explanatory power was improved for most of the interactions (see highlighted in Table 1 below) with the exception of the attributes related to attitude of health care worker. The LR test compares the log likelihoods of the two models and tests whether this difference is statistically significant (See Appendix 6)

Table 1. Log likelihood ratios for the mixed logit with interactions.

| Interaction terms | Log Likelihood ratio for generalized mixed model with interactions |
| --- | --- |
| **Conditional logit model** | **-3396.87** |
| **Generalized mixed logit** | **-2786.00** |
| Clean_Age. | **-2845.93** |
| Clean_Marr | **-3067.24** |
| Clean_Main | **-3691.09** |
| Medequip_Age | **-3861.89** |
| Medequip_Marr | **-3835.91** |
| Medequip_Main | **-3834.97** |
| Att_Age | **-2801.22** |
| Att_Marr | **-2786.00** |
| Att_Main | **-2786.00** |
| QualClin_Age | **-3834.90** |
| QualClin_Marr | **-3837.18** |
| QualClin_Main | **-3836.93** |

**DISCUSSION SECTION**

I think some of the discussion sections need to be revisited once the authors clarify the above questions related to methodology and results.

The entire discussion section was revised to correspond to the re-analysis of the data.

**Comment#1**

Last to second paragraph: Can the authors please elaborate the claim on “women with higher education levels have a greater understanding of the costs associated with delivery care”? I don’t think it’s a good idea to fit the interactions between costs and attributes. Moreover, the mixed effects results (Table 5) show that there is no significant preference heterogeneity for costs (as well as distance, medical equipment and supplies) then why fit interaction terms? Also, do the stratified analyses by the major sociodemographic variables provide similar results?

**Response to comment#1**

We re-ran the data and elaborated different claims with regard to the attributes. Since women with higher education have been identified in other settings to have strong preference for better quality delivery services in DCE’s conducted in similar neighboring sub-Saharan African countries such as Tanzania and Ethiopia, we focused our analysis on the sociodemographic variables of age, marital status and main income earner status. (Kruk, Paczkowski, Mbaruku, de Pinho, & Galea, 2009); (Kruk et al., 2010)

**Comment#2**

Can the authors please provide the stratified analysis results as the appendix for the readers to compare?

**Response to comment#2**

An appendix with the stratified analysis for the major sociodemographic variables has been included as an appendix 5

**Comment#3**

Last paragraph: I am very unclear about the authors’ main point here. In the Result section, the authors describe that “female headed households had a statistically significant disutility for high delivery costs”… first of all, it is very unclear what does “positive” utility coefficient means for the households headed by males. Do the authors suggest that male headed households (which seem the majority) prefer to have a higher cost, which then relieve some of the cost-related burden? Please be more specific how the preference of partners or being in male-headed households may affect women’s financial decisions or concerns

**Response to comment#3**

We revised the earlier assertions that had been made based on the previous interpretations of the data

**MINOR COMMENTS SECTION**

**Minor comment#1**

There are many grammatical errors and unclearly written sentences so I would recommend that this paper is to be carefully proofread.

**Response to minor comment#1**:

The manuscript has been carefully proofread and the grammatical errors and unclearly written sentences have been revised

**Minor Comment#2**

- Please check the references throughout the paper. It looks like the paper used a citation manager program, and some references did not properly show up. For example, in the first paragraph, the citations “((3); (4); (5);” should be “(3-5”); (UnitedNations, 2014) should be (X) with the correct reference number.

**Response to minor comment#2**

- The citations for 3-5 have been edited to be “(3-5)” as recommended
- The United Nations reference for 2014 has been revised to a numerical

**Minor Comment#3**

- 1st paragraph: “Rapid Urbanization” should be “Rapid urbanization”. Throughout the paper, there are many terms, which should not be capitalized when used in the middle of sentences.

**Response to minor comment#3**

- Rapid Urbanization has been changed to Rapid urbanization.
- The capitalizations in the middle of sentences have been revised

**Minor comments#4**

- 2nd paragraph in Introduction: “One study reported a maternal mortality ratio of 700 for every 1,000 births” -> this seems like an error. Do the authors mean 700 per every 100,000 births?
- 2nd para: “Kenyan Government abolished” -> “… removed” or “made delivery free of charge at public health…”
- 2nd Para. “However evidence”-> “However, evidence”

**Response to minor comments#4**

- The maternal mortality ratio has been adjusted 700 per every 100,000 births.
- The phrase Kenyan Government abolished  has been changed to “ made delivery free of charge at public health facilities”
- In the phrase  However evidence a comma has been inserted and this has been changed to However, evidence

**Minor Comment #5**

- Conditional model: the variable names are difficult to understand. Please define proper acronyms and refer to them.

**Response to minor comment#5**

**-** The variable names have been changed to proper acronyms for example Cleanliness is now clean. A legend has been created underneath the tables to refer to the acronyms that have been used.

**RESULTS**

**Minor comment#6**

- Please include percentages for those married and with secondary education in the text.

**Response to minor comment#6**

- The percentages for those married and with secondary education in the text as requested.

**Minor Comment#7**

- Table 3: “Planning for delivery” seems confusing. Does that refer to the previous pregnancies women had or for future pregnancy?

**Response to minor comment#7**

- The term “ planning for delivery” has been revised to refer to previous pregnancies

**Minor comment#8**

- Please define all acronyms in the footnote.

**Response to minor comment #8**

- All the acronyms have been included in the footnote.

**Minor comment#9**

- I recommend changing “Main earner status” to “Household head”, as that’s what the authors refer in the results and discussion. / Add “ago” after “Moved to the area over 5 years”

**Response to minor comment#9**

- “Main earner status” was a different variable from the “household head”. The main earner status assessed whether the woman is the main earner in the household> The household head assessed
- The word “ ago” has been added to the phrase “ Moved to the area over 5 years”

**Minor Comment#10**

- Table 6: it should be labelled as from “women in the mixed logit model”, not “women in the conditional model”

**Response to minor comment#10**

- The label for Table 6 has been changed to the “ women in the mixed logit model”

**REFERENCES**

(APHRC), (2012). Population and Health Dynamics in Nairobi's Informal Settlements Report of the Nairobi Cross-sectional Slums Survey(NCSS)2012.

Bohren, M. A., Hunter, E. C., Munthe-Kaas, H. M., Souza, J. P., Vogel, J. P., & Gulmezoglu, A. M. (2014). Facilitators and barriers to facility-based delivery in low- and middle-income countries: a qualitative evidence synthesis. *Reprod Health, 11*(1), 71. doi:10.1186/1742-4755-11-71

Hanson, K., McPake, B., Nakamba, P., & Archard, L. (2005). Preferences for hospital quality in Zambia: results from a discrete choice experiment. *Health Econ, 14*(7), 687-701. doi:10.1002/hec.959

Kenya National Bureau of Statistics, Ministry of Health/Kenya, National AIDS Control Council/Kenya, Kenya Medical Research Institute, Population, N. C. f., & Development/Kenya. (2015). *Kenya Demographic and Health Survey 2014*. Retrieved from Rockville, MD, USA: <http://dhsprogram.com/pubs/pdf/FR308/FR308.pdf>

Kruk, M. E., Paczkowski, M., Mbaruku, G., de Pinho, H., & Galea, S. (2009). Women's preferences for place of delivery in rural Tanzania: a population-based discrete choice experiment. *Am J Public Health, 99*(9), 1666-1672. doi:10.2105/ajph.2008.146209

Kruk, M. E., Paczkowski, M. M., Tegegn, A., Tessema, F., Hadley, C., Asefa, M., & Galea, S. (2010). Women's preferences for obstetric care in rural Ethiopia: a population-based discrete choice experiment in a region with low rates of facility delivery. *J Epidemiol Community Health, 64*(11), 984-988. doi:10.1136/jech.2009.087973

Larson, E., Vail, D., Mbaruku, G. M., Kimweri, A., Freedman, L. P., & Kruk, M. E. (2015). Moving Toward Patient-Centered Care in Africa: A Discrete Choice Experiment of Preferences for Delivery Care among 3,003 Tanzanian Women. *PloS one, 10*(8), e0135621. doi:10.1371/journal.pone.0135621

Mangham, L. J., Hanson, K., & McPake, B. (2008). How to do (or not to do) … Designing a discrete choice experiment for application in a low-income country. *Health policy and planning, 24*(2), 151-158. doi:10.1093/heapol/czn047

Oluoch-Aridi, J., Smith-Oka, V., Milan, E., & Dowd, R. (2018). Exploring mistreatment of women during childbirth in a peri-urban setting in Kenya: experiences and perceptions of women and healthcare providers. *Reprod Health, 15*(1), 209. doi:10.1186/s12978-018-0643-z

Tama, E., Molyneux, S., Waweru, E., Tsofa, B., Chuma, J., & Barasa, E. (2018). Examining the Implementation of the Free Maternity Services Policy in Kenya: A Mixed Methods Process Evaluation. *International Journal of Health Policy and Management, 7*(7), 603-613. doi:10.15171/ijhpm.2017.135

UnitedNations. (2014). *Population Facts*. Retrieved from New YorkAu:

WHO. (2016). *Standards for improving quality of maternal and newborn care in health facilities*. Retrieved from

Johnson R, Orme B. Getting the most from CBC. Sequim: Sawtooth Software Research Paper Series, Sawtooth Software; 2003. [[Google Scholar](https://scholar.google.com/scholar_lookup?title=Getting+the+most+from+CBC&author=R+Johnson&author=B+Orme&publication_year=2003&)]

Louviere, JJ., Hensher, D.A., SwaitJ.D., 2010 Stated Choice Mehtods: Analysis and Applications. Cambridge University Press.

Bliemer MCJ, Rose JM. Construction of experimental designs for mixed logit models allowing for correlation across choice observations. Transp Res B Methodol. 2010;44(6):720–734. doi: 10.1016/j.trb.2009.12.004
