# Supplementary material for: Eliciting women’s preferences for place of child birth at a peri-urban setting in Nairobi, Kenya: A discrete choice experiment
Source: PLoS One. 2020 Dec 10;15(12):e0242149. doi: 10.1371/journal.pone.0242149 (PMC7728449; doi:10.1371/journal.pone.0242149)
Supplement: Attachment — Submitted filename: Peri-urban Manuscript_PLOS ONE_FINAL_2822020.docx [file pone.0242149.s015.docx]

# **Eliciting women’s preferences for place of delivery at a peri-urban setting in Kenya: A discrete choice experiment**

Jackline Oluoch-Aridi^1, 2 *^ Mary B. Adam ^1, 3^ Francis Wafula^1^ and Gilbert K’okwaro ^1,^

1. Institute of Healthcare Management, Strathmore University, Nairobi, Kenya.

2. The Ford Family Program in Human Development Studies & Solidarity, Kellogg Institute of International Studies, University of Notre Dame.

3. Maternal Newborn Community Health, AIC hospital, Kijabe, Kenya

*Jackline.A.Oluoch-Aridi1@nd.edu

**Abstract**

Women’s selection of a place of delivery can affect maternal and newborn outcomes. Maternal and newborn mortality rates are high in peri-urban areas in Kenya, yet little is known about what drives women’s decisions on where to deliver. Understanding such factors can contribute to the development of patient-centered policies that reduce mortality. This study used the Discrete Choice Experiments (DCE) to quantify the relative importance of various attributes to the women’s decision on where to deliver at a peri-urban setting in Kenya. Participants were women aged 18-49 years, who had delivered at a health facility in the area. The DCE interviews were done on the Open Data Kit (ODK) platform at the women’s homes. To narrow down the number of choices, a D-efficient design was used to generate 16 choice-sets using JMP Pro software. Three alternatives of unlabeled choices were presented for each set; delivery at a facility A, delivery at a facility B, or none of the two (opt-out alternative). A conditional model was run to establish the relative strength of preferences. Sociodemographic variables of the women were interacted in a mixed logit model to assess how women’s preferences for selected attributes changed based on their characteristics. The DCE survey had a response rate of 87.5%. Health facility cleanliness was found to have the strongest association with choice of health facility (β=1.488 p<0.001) followed respectively by medical equipment and supplies availability (β= 1.435p<0.001). The opt-out alternative (β=1.424 p<0.001) came third. The attitude of the health care workers (β=1.347, p<0.001), quality of clinical services (β=0.385, p<0.00), distance to the health facility (β=0.339, p<0.001) and cost of delivery service (β= 0.0002 p<0.001) were 4^th^, 5^th^, 6^th^ and 7^th^ respectively. Further analyses suggested that there was preference heterogeneity, with women who were younger and were the main income earners having a stronger preference for clean health facilities. Older married women had stronger preference for availability of medical equipment and kind healthcare workers. Study findings show that women valued both technical and process indicators of quality of care. Understanding demand side barriers to access should inform policy on improvement of quality of care at health facilities in peri-urban areas in Kenya. DCE’s can help policy makers identify and rank what women value and lead to the development of people-centered strategies that take into account the preferences of women to improve maternal and newborn health outcomes.

**Key words:** women preferences, delivery services peri-urban, discrete choice experiment

**Introduction**

In 2015, an estimated 303,000 women were reported to have died in the developing world out of maternal causes.  Nearly all maternal deaths (99%) occur in developing countries with over half of these deaths occurring in sub-Saharan Africa (1).  This is a key reason behind Sustainable Development Goal 3 (SDG3), which aims to reduce the global maternal mortality ratio (MMR) to 70 for every 100,000 live births by 2030 (2). Skilled attendant delivery is considered an effective way of achieving this (3),(4) yet evidence suggests that it remains low, particularly across rural and poorer urban locations. The UN projects that by 2020, most Africans will be living in urban areas (5) Rapid urbanization has resulted in informal settlements, posing additional access challenges (5).

Kenya’s maternal mortality ratio of 362 per 100,000 live births is relatively high for a lower middle income country (6).   The ratios are particularly high across peri-urban informal settings. One study reported an MMR of 700 per 100,000 in two informal settlements in Nairobi (7).   The high rates have been attributed to inequities across peri-urban settings that residents within cities face (5). To improve maternal health outcomes, the Kenyan Government made delivery services free at public health facilities in 2013 (8).  Free delivery policy substantially reduced out of pocket costs leaving families to cover only incidental charges. Health centers and dispensaries received direct reimbursements through the hospital Sector Services Fund (2500 Ksh). Hospitals that offered referral care including C-sections were directly reimbursed through the Hospital Management Service Fund (Ksh 5000) (9). Women continued to pay incidental charges which varied by facility and presumably were for supplies that were not part of the hospital reimbursement package from the government.

Early evidence suggests that the policy may have increased facility delivery up from 86% to 95 % in five urban settings in Kenya (10). Another study reported an increase of 27% and 16% for deliveries and antenatal services across county referral hospitals and low-cost private facilities respectively (11).  On the other hand, studies showed that health systems preparedness was inadequate, posing danger of poor quality (12). Challenges reported included delays in health facility reimbursements for deliveries, lack of ambulances for referral, stock outs of essential supplies and health worker shortages (13-15). For that reason, there is increased awareness on the need to focus on both access and quality of service (14).

Quality is measured on the basis of safety, effectiveness, timeliness, efficiency, equity and patient-centeredness (15). The Lancet Commission on High Quality Health Systems estimated that one in three people in low and middle income countries (LMIC’s) have had a negative experience with the health system in the areas of attention, respect, communication and length of visits and disrespectful treatment (16).Evidence also indicates that poorer women, such as those in the peri-urban settings, have a higher likelihood of encountering poor quality of maternal health services in Kenya (17).

While there has been increased focus on quality (12), (13) efforts have mainly focused on health system inputs and satisfaction at the end of the continuum of care both of which fail to identify and rank the relative weight of demand side barriers to access (18). Such studies cannot fully explain what drives women to choose a facility. This information is particularly useful for prioritization in resource constrained settings. This is where Discrete Choice Experiments (DCE) have found meaningful use.

DCE’s allow health services users to state individual preferences based on predefined hypothetical choices. They are based on the assumption that services can be described by their attributes, and that the value of a service depends on the nature and level of these attributes (19). The theoretical basis for Discrete Choice Experiments DCEs is described elsewhere (19), (20). DCE’s have been used to examine a broad range of health system challenges in sub-Saharan Africa (21), patient preferences for hospital services (22) and maternal health services in rural areas of Tanzania and Ethiopia (23), (24), (25). The studies have mainly focused on women in rural settings. The few studies that have looked at more urban settings have focused on other determinants of delivery (26),(27),(28),(29) This study uses the DCE methodology to understand the attributes of the health system that women value most when making the decision on where to deliver.

# **Materials and methods**

**Study Setting**

The study was conducted at Embakasi-North, a sub-county in Nairobi County with a population of 181,388 people and is located about 10 km to the East of Nairobi City. Embakasi-North is home to Dandora, an area that houses the largest municipal dumpsite in Nairobi, and is characterized by low-income residential housing estates. The area is served by a mix of public, private and faith-based facilities of different levels. Mama Lucy Maternity Hospital, a secondary referral hospital, is located in the neighboring sub-county. Maternity facilities utilized by women in these informal settlements vary widely in terms of quality of care that they provide.

**Development of DCE attributes and attribute levels**

The study entailed conducting a literature review and doing a qualitative study to determine attributes and attribute levels that were important to women. The qualitative study sought to explore the perceptions and experiences of women visiting health facilities in the area. After obtaining informed consent from the women, trained facilitators led the focus group discussions (FGDs). Women were asked to explain how they made the choices of facility to deliver in and identify features that drove them to the facility. Each FGD had 6-8 women. In the end, 40 purposively selected women were interviewed. The characteristics of the women interviewed are contained in (Appendix 1).

Interview data were entered into Nvivo 11 and coding done. Thematic analysis was done following the six key steps, namely, familiarization with the data, coding, grouping codes, identifying themes, additional coding and refining of themes, and writing up the results. Four broad themes were identified: perceived quality of delivery services, financial access, physical amenities at the facility, and health workers strike. The themes helped in deriving attributes and attribute levels. The selected attributes were piloted on 30 women residing outside of the study setting in a neighboring sub-county to test for suitability and the cognitive response of the women in understanding the selected attributes.

**Table 1. List of attributes and attribute levels included for the DCE.**

| Attribute | Attribute level |
| --- | --- |
| Interpersonal treatment at the health facility | Good interpersonal treatment |
|  | Bad interpersonal treatment |
| Attitude of healthcare workers | Kind and supportive healthcare worker |
|  | Unkind and unsupportive healthcare worker |
| Availability of medical equipment and supplies | Medical equipment and supplies available |
|  | Medical equipment and supplies not available |
| Distance to the health facility | Health facility is close to residence |
|  | Health facility is far from residence |
| Cleanliness of the health facility | Clean health facility |
|  | Dirty health facility |
| Cost of delivery service  (Kenyan Shillings/ Ksh) | 3000; 5000; 8000 |

***Costs are not zero even with free delivery policy due to incidental fees charges at government facilities.**

The pilot showed that the attributes could be easily understood and traded-off by the women. Some attributes such as the costs of delivery attributes were revised and were chosen based on what the women reported they had paid when going to deliver. The costs ranged from 3000Ksh to 8000 Ksh for normal uncomplicated deliveries in both the public and private health facilities. The costs were inclusive of out-of-pocket costs that the women were charged during delivery. These costs were present even at facilities that had the “free delivery” policy. For a complete list of the attributes and attribute levels selected for the DCE, See Table 1.

**The DCE Experimental Design**

The study was designed as an unlabeled DCE with sixteen choice set presented under three alternatives: alternative of health facility A, alternative of health facility B, and an opt-out alternative where the woman would choose none of the two facilities, explained as a preference for home delivery. Figure 1 shows a sample choice-card with a scenario showing the final attributes and attribute levels included. Appendix 2 shows details of the original design.

All attributes in the choice experiment were dichotomous, except cost, which had three levels. This resulted in a design of (2^5^ x 1^3^) =96 = (96*95)/2 = 4560 possible choices in the full fractional design. A fractional factorial design helped to reduce the choices from 35 to 16, making it simpler for the respondents. This was done using a D-efficient design using Johnson and Orme method (30). We used JMP software for experimental design and resulted in a D-error of 0.3. (JMP Pro). All the attributes were dummy coded to allow comparison against a reference category. The D-efficient design also allowed for favorable design such as orthogonality, level balance, minimum balance and overlap (31) .The 16 choice-set questions were generated from the design. The choice-sets were grouped into two, and each woman answered eight questions in a single block

**Data collection for the household and the DCE survey**

Following administration of informed consent, a random sample of women of reproductive age (18-49 years) were recruited from a larger household survey in the area. The inclusion criteria was women who had delivered in the past five years. The main household questionnaire was a composite tool carrying questions from the Kenya Demographic Health Survey and the African Population and Health Research Survey (5) (6). The survey contained questions on women’s sociodemographic characteristics and maternal health services utilization variables. The questionnaire is attached in appendix 3, and details of the sampling process from the larger household survey provided in appendix 4. The sample size for the DCE was calculated using the Orme methodology (30). Those below 18 years were excluded.

The household survey was conducted between August and September 2017 by trained research assistants using Open Data Kit (ODK) platform (See Figure 2). This was followed by the DCE survey, which asked women to imagine a hypothetical scenario where they were expecting a baby and had to choose between facilities A and B for delivery (or none). The women were told that the opt-out option implied home delivery. They were also told that there were no right or wrong or right answers, and that they were free to stop the experiment at any time.

**Ethics approval**

Ethics approval for the study was provided by the African Medical Research Foundation (AMREF) research Committee, the National Commission for Science and Technology (NACOSTI) as well as the Country Directors of health in charge of the sub-county.

# **Data analysis**

The DCE data was analyzed using the Random Utility model (32), a model that expresses the utility ‘U’ in of an alternative *i* in a choice set *C_n_* (perceived by individual *n*) as two parts: 1) An explainable component specified as a function of the attributes of the alternatives *V (X_in_, β)*; and 2) an unexplainable component (random variation) *ε _in_*.

*U _in_* = *V* (*X_in_*, β) + ε *_in_*

The individual *n* will choose alternative *i* over other alternatives in a choice set C if and only if this alternative gives the maximized utility. The relationship between the utility function and the observed *k* attributes of the alternatives can be assumed under a linear-in-parameter function (31). Therefore, the utility the respondents attach is related to the attribute and attribute levels within the choice-sets, meaning that if alternative i is chosen within a choice set, i will yield the maximum utility compared to j alternatives. Α is the alternative specific constant, x are the attributes in the DCE and β are the coefficients describing the marginal utility of the attribute. The standard conditional logit model is below:

*V*_in_ = α_i_ + β_i_*x*_i1_ + … + β_k_ *x*_i + e_

The data were imported and analyzed in Stata 15 (StataCorp LP, College Station, USA). Descriptive statistics were calculated for the non-DCE variables. The cost attribute was assumed to be linear while all other attributes were categorical variables, therefore non-linear. A base conditional model was used to estimate the mean change in utility, preference which respondent placed on attributes (31).

α i is a constant term that represents the general preference for place of delivery at a health facility compared to the alternative of opting out and having a home delivery. Dummy coding was used for the data, each attribute level was assigned a value of 1 whenever it was retained and 0 when omitted. The cost of delivery service was entered in the model as a continuous variable. All the other five variables were coded as categorical variables. The Utility Model makes the assumption that women will trade-off between the different attribute levels and choose the alternative that gives the greatest utility. The conditional model is suitable for estimating average preferences across respondents. The utility function was estimated for the following model:

U_i_=  α_i_ + β1GoodQualClin + β2kindattitudeofhealthworkers + β3Medequipavail) _+_β4Shortdist + β5clean) + β6Costs + ε (error term)

α_i_ is the alternative specific constant (ASC) term that shows the preference for place of delivery (either a health facility or home), β’s 1-6 are the parameters for each of the attribute levels and ε is the error term.

β1= Good Quality of clinical services, β2 =kind attitude of the health care worker,

β3= availabilityofmedicalequipmentanddrugsupplies, β4= short distance to health facility, β5= cleanliness of the heath facility and, β6 Costs

The dependent variable is the place of delivery represented by the unlabeled choices A, B and the opt-out (home delivery), while the independent variables are the respective attribute levels of the characteristics of the place of delivery. The base conditional logit model assumed homogeneous preferences across respondents (31). The output of the conditional logit model contains the beta which shows the magnitude of the preferences for the attribute. Due to the assumption of irrelevant independent alternatives, the presence of heterogeneity in choices we estimated a generalized mixed logit model to assess for preference heterogeneity amongst the women (33). This was done by extending the generalized model and testing interactions between the sociodemographic and the women’s attributes in order to investigate how preferences may vary according to observed individual characteristics. The sociodemographic characteristics that were included as interaction terms include sociodemographic characteristics that have been known to influence place of delivery in Kenya were also included such as maternal age, marital status, education and income status (34), (35), (36), (37)

The output of the mixed logit model includes both the mean and the standard deviations of the random parameter estimates with confidence levels. The mean parameter estimate represents the relative utility of each attribute while the standard deviations for a random parameter suggest the existence of heterogeneity in the parameter estimates over the sampled population around the mean parameter estimate i.e., different individuals possess individual-specific parameter estimates that may be different from the sample population mean parameter estimates. The p-value of the interactions shows statistical significance for an interaction between sociodemographic variables and attributes hence signifying the influence of the woman’s characteristics. Insignificant parameter estimates for derived standard deviations indicate that the dispersion around the mean is statistically equal to zero, suggesting that all information in the distribution is captured within the mean. The theoretical validity of the design will be explored by examining the signs and significance of parameter estimates (31).

# **Results**

## **Participants’ characteristics**

A total of 481 women were selected for the interview. Of the women, 83% were married, 88% had secondary education and 52% had at least one child. Only 17% of the women said they were heads of their households. The participants’ characteristics are summarized in Table 2. A total of 421 (87.5%) did the DCE. Ten women did not complete the DCE exercise, reducing the sample for analysis to 411 women. The total number of observations analyzed for the DCE was 20,080.

**Table 2. Sociodemographic Characteristics of the women in the a peri-urban setting who were administered for DCE survey (N=421)**

| Sociodemographic variables | N (%) | |
| --- | --- | --- |
| Age n (mean(SD)) | 24 (0.2) |  |
| Marital status |  |  |
| Single | 68 | 17 |
| Married | 353 | 83 |
| Education |  |  |
| Primary School | 147 | 35 |
| Secondary School | 225 | 53 |
| University/tertiary | 49 | 12 |
| Parity |  |  |
| 1 | 251 | 52 |
| > 2 | 230 | 48 |
| Is the main earner |  |  |
| Woman not main earner | 366 | 87 |
| Woman is main earner | 55 | 13 |
| Head of Household education |  |  |
| Primary school | 66 | 16 |
| Secondary school | 187 | 44 |
| University/Some tertiary | 168 | 39 |
| Woman’s influence on decision making within the household |  |  |
| Woman had no influence in decision making | 54 | 13 |
| Woman had influence in decision making | 367 | 87 |

The majority of the participants (73%) had intended pregnancies and had planned to deliver at a health facility. Three-quarters reported attending the recommended four antenatal clinic visits. Nearly 77% had no health insurance cover. Only 7% said they were referred to a tertiary health facility for delivery. The health system utilization variable are summarized in Table 3.

**Table 3. Health system utilization variables for women in peri-urban setting women (N=421)**

| Health system utilization variables | N | (%) |
| --- | --- | --- |
| Pregnancy intentions |  |  |
| Pregnancy not intended | 113 | 27 |
| Pregnancy intended | 302 | 73 |
| Place of delivery |  |  |
| Public health facility | 235 | 41 |
| Private health facility | 159 | 59 |
| Place of delivery (level of delivery facility) |  |  |
| Home | 27 | 6 |
| Tertiary | 318 | 75 |
| Primary | 76 | 18 |
| Planning for delivery |  |  |
| Not planned health facility | 109 | 26 |
| Planned health facility | 311 | 74 |
| Number of ANC visits attended |  |  |
| ANC visits <4 | 182 | 43 |
| ANC visits >4 | 239 | 57 |
| Referral status |  |  |
| Not referred to a tertiary health facility | 390 | 93 |
| Referred to tertiary health facility | 31 | 7 |
| Specialist services |  |  |
| Did not see specialist | 355 | 84 |
| Saw a specialist | 66 | 16 |
| Whether the woman had a cesarean section |  |  |
| Didn’t deliver via cesarean | 355 | 84 |
| Had a cesarean section | 66 | 16 |
| Health Insurance status |  |  |
| Didn’t have health insurance | 325 | 77 |
| Had health insurance | 96 | 23 |
| When the woman moved to the peri-urban setting |  |  |
| Moved within the last 5 years | 218 | 52 |
| Moved to area over 5 years ago | 203 | 48 |

### **The Conditional model results**

The Conditional model results (Table 4) indicate that all attributes for place of delivery were statistically significant for all attributes, meaning that they were all valued by the women. The variables with the strongest association were cleanliness, availability of medical equipment and drugs, and the opt-out alternative (home delivery) in order of strength. Health worker attitude, quality of clinical services provided, distance and cost were fourth, fifth, sixth and seventh respectively. One finding that was rather unexpected was a positive coefficient for the cost variable, which would suggest that the women had a utility for high delivery costs.

**Table 4. Conditional Logit model for a discrete choice experiment assessing women’s preferences in a peri-urban setting in Kenya**

| **Attribute** | **β** | **S.E** | **P value** | **C.I** | **Expected sign** |
| --- | --- | --- | --- | --- | --- |
| Clean | 1.488 | 0.434 | <0.001 | (1.403-1.573) | + |
| Medequip | 1.435 | 0.046 | <0.001 | (1.343-1.527) | + |
| ASC (opt-out) | 1.424 | 0.134 | <0.001 | (1.162-1.685) | + |
| Attitude | 1.347 | 0.038 | <0.001 | (1.272-1.423) | + |
| Qualclin | 0.385 | 0.045 | <0.001 | (0.297-0.473) | + |
| Distance | 0.339 | 0.037 | <0.001 | (0.267-0.412) | + |
| Cost | 0.0002 | 0.0000135 | <0.001 | (0.0002- 0.00024) | - |
| No. of Observ. | 20208 |  |  |  |  |
| Pseudo R^2^ | 0.5410 |  |  |  |  |
| Wald Chi | 4564.83 |  |  |  |  |
| Prob >chi2 | 0.0000 |  |  |  |  |
| Log likelihood | -3396.87 |  |  |  |  |
|  |  |  |  |  |  |

**Legend** Clean- Cleanliness of the health facility, Medequip Medical equipment and drugs, ASC-Alternative Specific Constant, Attitude- Attitude of healthcare workers, Qualclin. - Quality of the clinical delivery services, Distance- Distance to the health facility

**The Generalized mixed logit model**

For the generalized mixed multinomial logit model with no interactions, all the mean coefficients values for all the attributes, including the opt-out, were statistically significant (Table 5). This meant that we could reject the null hypothesis that stated that the attributes selected were not important to the women respondents. However, the opt-out option had a lower significance level, implying that there was less variance in the characteristics of the respondents who chose this option. All the attributes had statistically significant parameter estimates for the standard deviation, except the attributes on cost and distance.  This implied that there was insufficient variation for individual specific parameter estimates that might be different from the sample population mean meaning all the information for the attribute of cost and distance was contained in the mean parameter.

**Mixed Logit model with interactions between sociodemographic variables and attributes**

Additional analyses sought to examine the interaction between preferred attributes and selected sociodemographic variables shown in previous studies to have an association with health facility use. These variables included age, secondary education, marital status and main earner status. All interactions between the attributes of cleanliness and all the interaction covariates were significant with the exception of marital status. The availability of medical equipment and drug supplies had statistically significant mean parameter estimates, with all covariates with the exception of marital status and main earner status. All the interactions for the interactions between the attitude of healthcare workers and the covariates were statistically significant at the 95% level. The only significant interaction was between quality of clinical care services with secondary education.

Younger unmarried women with a secondary education and who self-identified as main income earners in their household had a significant strong preference for clean health facilities. Older women with a secondary education showed a strong preference for a health facility with availability of medical equipment and supplies. All the interaction between the attitude of healthcare workers and age, marital status and main earner status had a statistically significant parameter estimate. Older women, married women with a secondary education and who identified themselves as main earners all showed a strong preference for a kind and supportive healthcare worker. Only women with secondary schooling showed a strong preference for quality of clinical care. Lastly all the interactions between the variables of cost and distance were statistically insignificant at the 95% level. See Table 5 below for the details on the base model (generalized mixed logit and the interactions model with the selected sociodemographic variables)

**Table. 5 mixed logit model results with and without interactions for a discrete choice experiment addressing facility preferences for delivery among women, in a peri-urban setting in Kenya**

|  | **Base model** | | **Interaction terms (Mean Parameter)**  **w/Sec educ** | | **w/ age** | | **w/ marital status** | | **w/main earner** | |
| --- | --- | --- | --- | --- | --- | --- | --- | --- | --- | --- |
| **Attribute** | **β^a^** | **p-value** | **β^a^** | **p-value** | **β^a^** | **p-value** | **β^a^** | **p-value** | **β^a^** | **p-value** |
| Medequip | 2.266* | <0.001 | 1.660* | <0.001 | 0.047* | <0.001 | 0.051 | 0.606 | 0.871 | <0.001 |
| Clean | 2.258* | <0.001 | 1.769* | <0.001 | -0.037* | 0.007 | -0.282* | <0.001 | 1.649 | <0.001 |
| Attitude | 2.039* | <0.001 | 1.681* | <0.001 | 0.124* | 0.001 | 1.155* | 0.001 | 0.246 | 0.470 |
| QualClin | 0.570* | <0.001 | 0.575* | <0.001 | -0.017* | <0.001 | -0.036 | 0.614 | -0.090 | 0.285 |
| Distance | 0.445* | <0.001 | 0.467* | <0.001 | 0.017* | <0.001 | 0.439* | <0.001 | 0.399 | <0.001 |
| Cost, Ksh^b^ | -8.091* | <0.001 | -8.166* | <0.001 | -11.271 | <0.001 | -8.102 | <0.001 | -8.262 | <0.001 |
| **Interaction terms (SDs)** |  |  |  |  |  |  |  |  |  |  |
| Medequip X covariate | - | - | 0.330* | <0.001 | 0.007* | 0.001 | 0.042 | 0.333 | 0.093 | 0.456 |
| Clean X covariate | - | - | 0.389* | 0.001 | 0.014* | 0.016 | 0.164 | 0.456 | 0.562* | 0.001 |
| AttitdeX covariate | - | - | 1.314* | <0.001 | 0.037* | 0.002 | 0.611* | 0.001 | 1.106* | 0.001 |
| QualClin X covariate | - | - | 0.698* | <0.001 | 0.0014 | 0.228 | -0.004 | 0.491 | 0.009 | 0.727 |
| Distance X covariate | - | - | 0.008 | 0.700 | -0.0001 | 0.860 | -0.0001 | 0.993 | -0.011 | 0.778 |
| Cost X covariate | - | - | 0.043 | 0.208 | 0.0013 | 0.986 | -0.031 | 0.378 | 0.07 | 0.679 |
| No. of respondents |  |  | 411 | 411 | 411 | 411 | 411 | 411 | 411 | 411 |
| No. of observations |  |  | 20,208 | 20,208 | 20,208 | 20,208 | 20,208 | 20,208 | 20,208 | 20,208 |
| Log-likelihood | -2831.69 |  |  |  |  |  |  |  |  |  |
| Prob> χ2 | 0.0000 |  |  |  |  |  |  |  |  |  |
| Likelihood ratio χ2 | 80646.83 |  |  |  |  |  |  |  |  |  |

**Model estimates for preference for attribute levels**

The results showed that both models (conditional logit and mixed logit) were statistically significant (p < 0.05). The chi squared tests were also significant for both models (p<0.0001). The log pseudo likelihood of the conditional multinomial logit was -3396.87 and the generalized mixed conditional model was -2813.69. The models with interactions provided improved explanatory power for the generalized mixed logit model. (See Appendix 6).

**Validity of the data**

The theoretical validity was also checked through comparing the expected direction of the parameter estimates and this was found consistent with expectations with the exception of the attribute on costs, which had a positive coefficient. One would generally expect it to have a negative one because this signifies a utility for lower costs for delivery services. It did have a negative coefficient in the mixed logit analyses.

## **Discussion**

To the best of our knowledge, this study is the first DCE study conducted in a peri-urban setting in sub-Saharan Africa.  Previous studies in sub-Saharan Africa such as those done in and Zambia, Tanzania, and Ethiopia were conducted in rural settings (22-25). The study found that all the attributes had an effect on the decision on where to deliver. The women in this setting highly valued aspects of quality related to the technical quality such as the cleanliness of the health facility and the availability of equipment and supplies. They also valued aspects related to processes involved in delivery, particularly, the attitude of healthcare workers and to a lesser extent clinical quality during delivery. Studies using other methods done in maternity settings in urban Kenya have identified similar factors associated with satisfaction, including waiting time, attitude of the health providers, availability of drugs, affordability of services, staffing level and cleanliness (38).

The most valued attribute was health facility cleanliness, suggesting that relatively less costly interventions such as facility hygiene may increase skilled attendant deliveries in such settings. Yet, studies have shown hygiene to be poor across facilities in informal settlements (27). The Kenya Quality Model for Health (KQMH), the Kenyan government’s quality management framework, emphasizes hygiene as a primary intervention across facilities (39). Poor hygiene may cause users to avoid a facility and travel longer distances for care, which can cause delays and worsen maternal indicators. Such behavior has been documented in Tanzania for instance (40). Cleanliness has also been identified as an important attribute in Ethiopia and in Zambia (22, 23).

The second most highly ranked attribute was availability of medical equipment and drug supplies. This was also reported in Ethiopia (23). A recent study in Kenya found that only two of five health centers assessed had acceptable emergency obstetric care capability(41). Additionally recent assessments at Kenyan health facilities found that essential medical equipment and drug supplies were unavailable at 31% at public health facilities 59% of private health facilities (18).  However evidence shows that availability of infrastructure such as equipment may not necessarily translate into effective coverage for obstetric complications (42).

The kind and supportive attitude of healthcare workers was ranked third. Similar findings have been reported across Africa, including Zambia, Tanzania and Ethiopia (22, 23, 24). A recent review reported that the attitude of health workers managing women during labor and delivery presented a major quality challenge across low income settings (43). Similar findings have been reported locally (44),(45) (46) and other African countries (47). As a result, there has been more emphasis on promoting accountability for the actions of health care workers with regard to mistreatment of women during delivery (48),(49).

When attributes were interacted with sociodemographic characteristics that are known to influence women’s preferences for health facility for delivery, we found that younger unmarried women with a secondary education who were main earners had a stronger preference for clean health facilities. As expected women with a secondary education also had a strong preference for health facilities with medical equipment and supplies, health workers with a kind attitude and good quality care by health care workers The preference is consistent with local literature and other low-income countries with secondary educated women preferring to delivery in health facilities that signal high quality (36), (50). Peri-urban settings are increasingly populated with women with secondary education and it is important for the health system to be responsive to their preferences (6).

Older women on the other hand expressed the strongest preference for health facilities with medical equipment and supplies. Reasons are not clear. It may be that older women have more experience with the health system and have higher expectations. This then drove them to choose health facilities that had these types of equipment. Older women might also strongly rely on social networks for decision making on place of delivery (51)

Only cost and distance did not have significant preference heterogeneity. This implies that there was no variation in the characteristics of individual women who chose these attributes. Attributes such as distance that have been reported as important attributes in certain contexts appeared to have lower value across the study setting, possibly because the women could geographically access several healthcare facilities easily.  A rural setting DCE study reported distance to be lowly ranked attribute (23). The low effect of distance may partly explain why attributes that signal quality such as cleanliness were the most important (23). Policy may build on this observation to push for quality improvement at healthcare facilities, although the concern is that less visible aspects of quality may be ignored.

**Strengths and limitations of the study**

The main limitation of the study was the challenge in developing certain attributes accurately. This might have led to the underestimation of the parameter estimates such as the cost leading to a positive coefficient instead of a negative. Also, difficulty in definition of the attribute on quality of clinical services led to a relatively weak specification, mainly because the translation of the word ‘treatment’ carries both clinical and non-clinical (client relation) connotation to the women. The women may not have been able to separate the two. The gold-standard for assessing clinical quality is measuring staff adherence to guidelines, something that was beyond the scope of this study. However, effort was made to improve the theoretical validity of the study through doing the qualitative study to help identify new attributes of importance in the context, or confirm the ones identified in literature.

**Conclusion**

In conclusion, understanding the relative contribution of factors that influence the choice of a place for delivery is important for policy makers who are aimed at reducing maternal and newborn deaths as well as improving the quality of care. In exploring the complex context of facility based delivery for women it is important for policy makers to have a deeper understanding on women’s preferences this will help in overcoming barriers to high quality delivery care and structuring more patient-centered services that can lead to improved maternal health outcomes.

**Supporting information**

S1 Appendix. The Characteristics of women interviewed in the Focus Group Discussions

S2 Appendix. The DCE Experimental design

S3 Appendix. The Household survey questionnaire.

S4 Appendix. Sampling for the main household survey.

S5 Appendix. Dataset for the DCE and household survey for women in the peri-urban setting.

S6 Appendix Log likelihood ratio calculations

S7Additional analysis for the Mixed Logit model with interactions

S7 Appendix. Ethical approval form.

S8 Appendix. Informed consent form.

**Acknowledgments**

The authors are first of all grateful to the women and healthcare workers living in Embakasi-North for allowing us to interview them. We would like to thank our research assistants; Cindy Mical, Brian Ambutsi, Christine Achieng and Mercy Ngao. We appreciate Melvin Obadha, Maurice Baraza and Sydney Oluoch for assisting with the data analysis. We thank the Institute for healthcare Management PhD seminar group consisting of Dr. Ben Ngoye, Tecla Kivuli and Eric Tama for their critical feedback during PhD seminars.

**Author contributions**

**Conceptualization:** Jackline O. Aridi **Formal analysis:** Jackline O.Aridi

**Investigation:** Jackline O. Aridi

**Writing- original draft:** Jackline O. Aridi, Gilbert Kokwaro, Mary B. Adam and Frank Wafula

**Writing- review & editing:** Jackline O. Aridi, Gilbert Kokwaro, Mary B. Adam and Frank Wafula

**Ethical Approval:** Ethical approval to conduct this study was obtained from the AMREF Health-ESRC (P388/2017) and the permit to conduct the study was obtained from the National Commission for Science, Technology and Innovation (NACOSTI/P/17/34367)

**Consent for publication**

Not applicable

**Availability of data and material**

The deidentified data has been attached in the appendix S5

**REFERENCES**

1.	Alkema L, Chou D, Hogan D, Zhang S, Moller AB, Gemmill A, et al. Global, regional, and national levels and trends in maternal mortality between 1990 and 2015, with scenario-based projections to 2030: A systematic analysis by the un Maternal Mortality Estimation Inter-Agency Group. Lancet [Internet]. 2016;387(10017):462–74. Available from: http://dx.doi.org/10.1016/S0140-6736(15)00838-7

2.	Transforming our world: the 2030 agenda for sustainable development, United Nations General Assembly 2015 .

3.	World Health Organization department of reproductive health and research. Making pregnancy safer: the critical role of the skilled attendant. A joint statement by WHO, ICM and FIGO. Geneva, Switz WHO [Internet]. 2004;1–18. Available from: http://whqlibdoc.who.int/publications/2004/9241591692.pdf

4.	Tomedi A, Tucker K, Mwanthi MA. A strategy to increase the number of deliveries with skilled birth attendants in Kenya. Int J Gynecol Obstet. 2013;120(2):152–5.

5.	African Population and Health Research Center (APHRC). Population and Health Dynamics in Nairobi’s Informal Settlements: Report of the Nairobi Cross-Sectional Slums Survey (NCSS) 2012. Nairobi: APHRC. 2014;(April):1–185.

6.	National Bureau of Statistics Nairobi K. Kenya Demographic and Health Survey 2014 Key Indicators [Internet]. 2015. Available from: www.DHSprogram.com.

7.	Ziraba AK, Madise N, Mills S, Kyobutungi C, Ezeh A. Maternal mortality in the informal settlements of Nairobi city: What do we know? Reprod Health. 2009;6(1).

8.	Bourbonnais N. Implementing Free Maternal Health Care in Kenya. Kenya Natl Comm Hum Rights [Internet]. 2013;(November):3. Available from: http://www.knchr.org/Portals/0/EcosocReports/Implementing Free Maternal Health Care in Kenya.pdf

9.	Wamalwa EW. Implementation challenges of free maternity services policy in kenya: The health workers’ perspective. Pan Afr Med J. 2015;22:1–5.

10.	Calhoun LM, Speizer IS, Guilkey D, Bukusi E. The Effect of the Removal of User Fees for Delivery at Public Health Facilities on Institutional Delivery in Urban Kenya. Matern Child Health J. 2018 Mar 1;22(3):409–18.

11.	Njuguna J, Kamau N, Muruka C. Impact of free delivery policy on utilization of maternal health services in county referral hospitals in Kenya. BMC Health Serv Res. 2017;17(1):1–6.

12.	Tama E, Molyneux S, Waweru E, Tsofa B, Chuma J, Barasa E. Examining the implementation of the free maternity services policy in Kenya: A mixed methods process evaluation. Int J Heal Policy Manag [Internet]. 2018;7(7):603–13. Available from: https://doi.org/10.15171/ijhpm.2017.135

13.	Lang’at E, Mwanri L. Healthcare service providers’ and facility administrators’ perspectives of the free maternal healthcare services policy in Malindi District, Kenya: A qualitative study. Reprod Health. 2015;12(1):1–11.

14.	Owili PO, Muga MA, Mendez BR, Chen B. Quality of maternity care and its determinants along the continuum in Kenya: A structural equation modeling analysis. PLoS One. 2017;12(5).

15.	WHO. Standards for improving quality of maternal and newborn care in health facilities. World Heal Organ [Internet]. 2016;73. Available from: http://www.who.int/iris/handle/10665/249155

16.	Kruk ME, Gage AD, Arsenault C, Jordan K, Leslie HH, Roder-DeWan S, et al. High-quality health systems in the Sustainable Development Goals era: time for a revolution. Lancet Glob Heal. 2018;6(11):e1196–252.

17.	Sharma J, Leslie HH, Kundu F, Kruk ME. Poor quality for poor women? Inequities in the quality of antenatal and delivery care in Kenya. PLoS One. 2017;12(1):1–14.

18.	Chen A, Dutta A, Maina T. Primary Healthcare evidence from the PETS-PLUS survey policy brief . 2014;(July).

19.	Mangham LJ, Hanson K, McPake B. How to do (or not to do)...Designing a discrete choice experiment for application in a low-income country. Health Policy Plan. 2009;24(2):151–8.

20.	Ryan M, Farrar S. Using conjoint analysis to elicit preferences for health care. Br Med J. 2000;320(7248):1530–3.

21.	Honda A, Ryan M, Van Niekerk R, McIntyre D. Improving the public health sector in South Africa: Eliciting public preferences using a discrete choice experiment. Health Policy Plan. 2015;30(5):600–11.

22.	Hanson K, McPake B, Nakamba P, Archard L. Preferences for hospital quality in Zambia: Results from a discrete choice experiment. Health Econ. 2005 Jul;14(7):687–701.

23.	Kruk ME, Paczkowski M, Mbaruku G, De Pinho H, Galea S. Women’s preferences for place of delivery in rural Tanzania: A population-based discrete choice experiment. Am J Public Health. 2009;99(9):1666–72.

24.	Kruk ME, Paczkowski MM, Tegegn A, Tessema F, Hadley C, Asefa M, et al. Women’s preferences for obstetric care in rural Ethiopia: A population-based discrete choice experiment in a region with low rates of facility delivery. J Epidemiol Community Health. 2010;64(11):984–8.

25.	Larson E, Vail D, Mbaruku GM, Kimweri A, Freedman LP, Kruk ME. Moving toward patient-centered care in Africa: A discrete choice experiment of preferences for delivery care among 3,003 Tanzanian women. PLoS One [Internet]. 2015;10(8):1–12. Available from: http://dx.doi.org/10.1371/journal.pone.0135621

26.	Fotso JC, Ezeh A, Madise N, Ziraba A, Ogollah R. What does access to maternal care mean among the urban poor? Factors associated with use of appropriate maternal health services in the slum settlements of Nairobi, Kenya. Matern Child Health J. 2009;13(1):130–7.

27.	Bazant ES, Koenig MA. Women’s satisfaction with delivery care in Nairobi’s informal settlements. Int J Qual Heal Care. 2009;21(2):79–86.

28.	Izugbara CO, Kabiru CW, Zulu EM. Urban Poor Kenyan Women and Hospital-Based Delivery. Public Health Rep. 2009;124(4):585–9.

29.	Essendi H, Mills S, Fotso JC. Barriers to formal emergency obstetric care services’ utilization. J Urban Heal. 2011;88(SUPPL. 2):356–69.

30.	Orme BK. Sample Size Issues for Conjoint Analysis. Get Started with Conjoint Anal Strateg Prod Des Pricing Res [Internet]. 2010;57–66. Available from: https://www.sawtoothsoftware.com/download/techpap/samplesz.pdf

31.	Louviere JJ, Pihlens D, Carson R. Design of discrete choice experiments: A discussion of issues that matter in future applied research. J Choice Model [Internet]. 2011;4(1):1–8. Available from: http://dx.doi.org/10.1016/S1755-5345(13)70016-2

32.	Manski CF, The S, Journal S, Jun N, Manski CE. Daniel McFadden and the Econometric Analysis of Discrete Choice Published by : Wiley on behalf of The Scandinavian Journal of Economics Linked references are available on JSTOR for this article : Daniel McFadden and the Econometric Analysis of Discrete Ch. 2017;103(2):217–29.

33.	Hole AR 2008 Modelling heterogeneity in patients' prefferences for the attributes of a general practitioner appointment .J Health Econ 28:598-610 doi: 10.1016/j.jhealeco.2008.11.003. PMID: 18179837Nhs E. CHE Research Paper 15. 2001;

34.	Karanja S, Gichuki R, Igunza P, Muhula S, Ofware P, Lesiamon J, et al. Factors influencing deliveries at health facilities in a rural Maasai Community in Magadi sub-County, Kenya. BMC Pregnancy Childbirth. 2018;18(1):1–11.

35.	Kitui J, Lewis S, Davey G. Factors influencing place of delivery for women in Kenya: An analysis of the Kenya demographic and health survey, 2008/2009. BMC Pregnancy Childbirth [Internet]. 2013;13(1):1. Available from: BMC Pregnancy and Childbirth

36.	Gitimu A, Herr C, Oruko H, Karijo E, Gichuki R, Ofware P, et al. Determinants of use of skilled birth attendant at delivery in Makueni, Kenya: A cross sectional study. BMC Pregnancy Childbirth. 2015 Feb 3;15(1).

37.	Banke-Thomas A, Banke-Thomas O, Kivuvani M, Ameh CA. Maternal health services utilisation by Kenyan adolescent mothers: Analysis of the Demographic Health Survey 2014. Sex Reprod Healthc. 2017;12:37–46.

38.	Wandera Nyongesa M, Onyango R, Kakai R. Determinants of clients ’ satisfaction with healthcare services at Pumwani Maternity Hospital in Nairobi - Kenya. Int J Soc Behav Sci. 2014;2(1):11–7.

39.	Ministry of Health (2014) Kenya Quality Model FOR HEALTH (KQMH) : A training course for the health sector( facilitators manual) .

40.	Kruk ME, Hermosilla S, Larson E, Mbaruku GM. Bypassing primary care clinics for childbirth: a cross-sectional study in the Pwani region, United Republic of Tanzania. Bull World Health Organ. 2014;92(4):246–53.

41.	Echoka E, Makokha A, Dubourg D, Kombe Y, Nyandieka L, Byskov J. Barriers to emergency obstetric care services: accounts of survivors of life threatening obstetric complications in Malindi District, Kenya. Pan Afr Med J. 2014;17(Supp 1):4.

42.	Leslie HH, Sun Z, Kruk ME. Association between infrastructure and observed quality of care in 4 healthcare services: A cross-sectional study of 4,300 facilities in 8 countries. PLoS Med. 2017;14(12):1–16.

43.	Bohren MA, Hunter EC, Munthe-Kaas HM, Souza JP, Vogel JP, Gülmezoglu AM. Facilitators and barriers to facility-based delivery in low- and middle-income countries: A qualitative evidence synthesis. Reprod Health. 2014;11(1):1–17.

44.	Oluoch-Aridi J, Smith-Oka V, Milan E, Dowd R. Exploring mistreatment of women during childbirth in a peri-urban setting in Kenya: Experiences and perceptions of women and healthcare providers. Reprod Health. 2018;15(1):1–14.

45.	Abuya T, Warren CE, Miller N, Njuki R, Ndwiga C, Maranga A, et al. Exploring the prevalence of disrespect and abuse during childbirth in Kenya. PLoS One. 2015;10(4):1–14.

46.	Warren CE, Njue R, Ndwiga C, Abuya T. Manifestations and drivers of mistreatment of women during childbirth in Kenya: Implications for measurement and developing interventions. BMC Pregnancy Childbirth. 2017;17(1):1–14.

47.	Jewkes R, Abrahams N, Mvo Z. Why do nurses abuse patients? Reflections from South African obstetric services. Soc Sci Med. 1998;47(11):1781–95.

48.	Jewkes R, Penn-Kekana L. Mistreatment of Women in Childbirth: Time for Action on This Important Dimension of Violence against Women. PLoS Med. 2015;12(6):6–9.

49.	Afulani PA, Moyer CA. Accountability for respectful maternity care. Lancet [Internet]. 2019;394(10210):1692–3. Available from: http://dx.doi.org/10.1016/S0140-6736(19)32258-5

50.	Gabrysch S, Campbell OMR. Still too far to walk: Literature review of the determinants of delivery service use. BMC Pregnancy Childbirth. 2009 Aug 11;9:34.

51.	Moyer CA, Adongo PB, Aborigo RA, Hodgson A, Engmann CM, Devries R. “it’s up to the woman’s people”: How social factors influence facility-based delivery in Rural Northern Ghana. Matern Child Health J. 2014;18(1):109–19.
