# Supplementary material for: Eliciting women’s preferences for place of child birth at a peri-urban setting in Nairobi, Kenya: A discrete choice experiment
Source: PLoS One. 2020 Dec 10;15(12):e0242149. doi: 10.1371/journal.pone.0242149 (PMC7728449; doi:10.1371/journal.pone.0242149)
Supplement: Attachment — Submitted filename: Response to Reviewers_PLOSONE_7thOct2020.docx [file pone.0242149.s016.docx]

**Review Comments to the Author**

Please use the space provided to explain your answers to the questions above. You may also include additional comments for the author, including concerns about dual publication, research ethics, or publication ethics. (Please upload your review as an attachment if it exceeds 20,000 characters)

**Reviewer #2:**

**Comment**

1.The manuscript did not follow the journal guideline, therefore requesting the authors to revisit the guideline and improve it.

**Response:** The authors have reviewed the journal guidelines and followed them in an attempt to improve the manuscript. The abstract has been divided in the four sections of objective, methods, results and conclusion. The abstract word count of 300 words has been adhered to. All other areas that did not follow journal guidelines have been adjusted appropriately.

2.The in-text citation needs reworking, the brackets used are the same as the brackets used for none citation area for example abbreviations used () and in-text citation used (); authors could differentiate these by using [] in in-text citation. Also, several citations used for one information to be under one bracket and not each on its own bracket.

**Response:** The in-text citations have been reworked and the brackets used for the in-text citations have been distinguished between the other brackets used for non-citation. Several citations have been put under one bracket as advised.

3. In the experiment it was only one attribute which was objective others were subjective. Other attributes were very subjective; dirtiness, availability of equipment, distance. It is not mentioned on whether they were defined to the respondents or not.

**Response:** We appreciate the comment. Only one attribute was objective. We have included a sentence that illustrates how the subjective attributes were defined. These definitions were presented to the women on the choice-card. Women had the definitions prior to making their choices and this is stated in lines (157-159) See below

The attributes of the health facility were explained to the women using a choice-card that contained a brief description of the definition of the attributes. For example. Cleanliness meant a health facility that had a clean ward with clean beds, bathrooms and toilets (See Appendix 3). We have included an appendix 3 that shows the choice card that was shown to the women and how the subjective attributes were defined to the women to have a shared understanding.

4. I happened to review this paper submitted BMJ with manuscript number bmjopen-2020-038865, can authors explain why double submission?

**Response:** The study was part of a two-site study the BMJ manuscript presents the results of the Discrete Choice Experiment in Naivasha sub-County (named rural sub-County). The current manuscript reports on the results of the Discrete Choice Experiment within a peri-urban setting Embakasi-North sub County. We previously attempted to submit the results as a comparative study but was rejected and advised to handle the two sites separately. This is primarily because the attributes that were identified were different in each site and hence deemed incomparable directly. All five attributes were common for both sites with the exception of one attribute- cleanliness of the health facility in the peri-urban site and the availability of referral services in rural sub-County.

**Reviewer #3**

The authors have addressed all of these comments. The manuscript has improved significantly. I have no further comments.

**Reviewer #4**

Thank you very much for reviewing this manuscript. This article describes preferences of Kenyan women when making choices about the place of birth. It is an interesting and relevant topic and the results give insight in important attributes for women when choosing a place of birth. This knowledge is important to provide woman-centered care and to improve maternal and newborn outcomes. The previous reviewer assessed the manuscript very accurately and I think the researchers addressed these comments properly. These changes and additions contribute positively to the readability of the manuscript.

However, after reading this manuscript I have some additional comments and questions:

1. I would like to discuss the term ‘place of delivery’. To my opinion, women do not deliver a baby, but give birth to a baby. So, I would recommend to use the term ‘place of birth’ instead of ‘place of delivery’. I think this is more consistent with international literature in this area.

**Response:** Thank you for your comments and your recommendation for consistency with international literature. We will change the term from “place of delivery to ‘place of child birth’

2. Line 157: the word ‘right’ is in the sentence twice.

**Response:** The word right has been deleted and only one ‘right’ is maintained

3. Participants’ characteristics: this paragraph is not well structured and is difficult to understand. It seems that the percentages presented in line 234 and 235 belong to the study population who was selected for the interview (n=481).

However, table 2 presents the same percentages and the number of participants in table 2 is 421. This is unclear and confusing, especially because the number of women who completed the DCE-questionnaire is 411. These women are suitable for the DCE-analysis.
I think you have to present the sociodemographic results of the total study population who completed the DCE-questions (n=411) in table 2 and 3.

Do you have any information about the women who declined participation (n=60) or who did not complete the DCE-questionnaire (N=10).

**Response:**

The paragraph on participants characteristics has been revised for clarity. We have revised the participant characteristics to be reflective of the sub-sample that actually completed the DCE N=411.

We conducted additional analysis on the women who declined participation n=60 and found that they had no significant differences between them and the participants in the study. (N=60)

There was also no difference between the women who completed and those that did not complete the DCE questionnaire. (N=10)

The women were dropped automatically for having dominant choices.

4. In table 2: The numbers presented at parity =1 and parity >2 count to 481. I think this is not correct. These numbers have to count to 411.

**Response:** This comment is appropriate. The analysis has been revised to have the parity count = 411 taking into account only those women who completed the DCE questions

5. Table 2: Parity > 2 has to be parity ≥ 2 (gave birth 2 times or more)

**Response**: The parity variable is Table 2 has been revised to be parity ≥ 2 (gave birth 2 times or more)

**Reviewer #5**

This manuscript investigates women’s preferences for place of delivery in a peri-urban setting in Kenya using a discrete choice experiment. I have below comments and questions.

Please ask an English editor to edit the writing.

**Response**: An English editor has edited the writing as requested.

Line 133, Appendix 2 does not have contents for design. But Appendix 2 shows significant correlations (p<0.0001) between a couple of attributes. Please discuss if their correlations would affect the model fitting and results.

**Response:** The results were reviewed and we found that the correlations do not affect the model fitting or the results

Line 136, “(2^5 x 1^3) =96 = (96*95)/2 = 4560”, how can they equal with each other? 1^3 should be 3^1. They should be described clearly by separating the two parts of calculations.

**Response**: The two parts of the calculation was separated as follows

“(2^5 x 3^1) =96. The two alternatives choice-sets were calculated as follows (96*95)/2 = 4560”,

Line 136, There are only three choices for delivery places. To avoid confusion, ”the number of possible choices” may be said as “the number of alternatives of attribute levels”.

**Response**: the number of possible choices has been revised to “the number of alternatives of attribute levels”.

Line 137, where dose 35 come from?

**Response**: The 35 was an error and has been replaced with 16 which comes from the JMP software results that automatically calculated the results.

Line 142, how do you group the choice-sets? What eight questions in each group?

**Response:** This is randomly determined by the software that we use open data kit (ODK). The sentence has been revised to include this detail. The choice-sets were grouped into two through a process called blocking. This is a standard process that is used to reduce the burden of answering many questions for each woman using the ODK software.

In the results, for the statement such as “statistically significant/insignificant at the 95% level” on page 14, do you mean the significance level was set as 95%? If the type I error was set at 0.05, the significance level should be stated as 5%.

**Response:** Yes, we meant that the significance was set at 95% and the significance is at the 5% level. This wording has been changed to reflect significance is at the 5%level.

For the generalized mixed multinomial logit model with no interactions, all the mean coefficients values for all the attributes, including the opt-out, were statistically significant at the 5% level

Line 405-414, It is hard to find the matched supporting information.

**Response:** An attempt has been made to match the supporting information and all the supporting information is included in the Appendices as listed.
